# Deep Soil Layers of Drought-Exposed Forests Harbor Poorly Known Bacterial and Fungal Communities

**DOI:** 10.3389/fmicb.2021.674160

**Published:** 2021-05-07

**Authors:** Beat Frey, Lorenz Walthert, Carla Perez-Mon, Beat Stierli, Roger Köchli, Alexander Dharmarajah, Ivano Brunner

**Affiliations:** Forest Soils and Biogeochemistry, Swiss Federal Institute for Forest, Snow and Landscape Research WSL, Birmensdorf, Switzerland

**Keywords:** bacterial and fungal communities, deep forest soil layers, drought, *Fagus sylvatica*, FUNGuild, Illumina MiSeq sequencing, *Quercus* spp.

## Abstract

Soil microorganisms such as bacteria and fungi play important roles in the biogeochemical cycling of soil nutrients, because they act as decomposers or are mutualistic or antagonistic symbionts, thereby influencing plant growth and health. In the present study, we investigated the vertical distribution of the soil microbiome to a depth of 2 m in Swiss drought-exposed forests of European beech and oaks on calcareous bedrock. We aimed to disentangle the effects of soil depth, tree (beech, oak), and substrate (soil, roots) on microbial abundance, diversity, and community structure. With increasing soil depth, organic carbon, nitrogen, and clay content decreased significantly. Similarly, fine root biomass, microbial biomass (DNA content, fungal abundance), and microbial alpha-diversity decreased and were consequently significantly related to these physicochemical parameters. In contrast, bacterial abundance tended to increase with soil depth, and the bacteria to fungi ratio increased significantly with greater depth. Tree species was only significantly related to the fungal Shannon index but not to the bacterial Shannon index. Microbial community analyses revealed that bacterial and fungal communities varied significantly across the soil layers, more strongly for bacteria than for fungi. Both communities were also significantly affected by tree species and substrate. In deep soil layers, poorly known bacterial taxa from *Nitrospirae, Chloroflexi, Rokubacteria, Gemmatimonadetes*, *Firmicutes* and GAL 15 were overrepresented. Furthermore, archaeal phyla such as *Thaumarchaeota* and *Euryarchaeota* were more abundant in subsoils than topsoils. Fungal taxa that were predominantly found in deep soil layers belong to the ectomycorrhizal *Boletus luridus* and *Hydnum vesterholtii*. Both taxa are reported for the first time in such deep soil layers. Saprotrophic fungal taxa predominantly recorded in deep soil layers were unknown species of *Xylaria*. Finally, our results show that the microbial community structure found in fine roots was well represented in the bulk soil. Overall, we recorded poorly known bacterial and archaeal phyla, as well as ectomycorrhizal fungi that were not previously known to colonize deep soil layers. Our study contributes to an integrated perspective on the vertical distribution of the soil microbiome at a fine spatial scale in drought-exposed forests.

## Introduction

The significance of soil microorganisms is receiving increasing attention, as they are relevant drivers of ecosystem functions (Bardgett and van der Putten, 2014). Soil microorganisms such as bacteria and fungi play important roles in the biogeochemical cycling of soil nutrients because they act as decomposers or are mutualistic or antagonistic symbionts, thereby influencing the growth and health of other organisms (Rousk and Bengtson, 2014). In addition, they emit greenhouse gases and, thus, affect the climate globally (Oertel et al., 2016; Jansson and Hofmockel, 2020). However, most studies have been focused primarily on the top 20 cm of soil, where microbial biomass, activity and diversity are assumed to be the greatest. In contrast, little is known about the microbiome in the soil subsurface (Jiao et al., 2018), although trees in forest ecosystems have roots down to 2 m or more (Schenk and Jackson, 2002).

In general, living conditions for soil organisms and roots are harsher in deeper soil layers, where the soil density is higher, oxygen concentrations are lower, and carbon and nutrients are less available (Lennon, 2020). Carbon and nutrients typically are more available in the upper soil, mainly due to larger quantities of leaf and fine root litter and root exudates, as well as more biotic activity (Eldridge et al., 2016; Liu et al., 2020). Consequently, microbial biomass is usually one to two orders of magnitude lower in the subsoil than in the topsoil (Eilers et al., 2012; Spohn et al., 2016). Nevertheless, the cumulative biomass of bacteria and fungi inhabiting deeper soil horizons can be as high as in the topsoil, and these microorganisms play similarly important roles in biogeochemical processes, including soil carbon and nitrogen cycling, soil formation, iron redox reactions, and pollutant degradation (Brewer et al., 2019). Some groups of microorganisms appear predominantly in deeper soil horizons, such as the candidate phylum *Dormibacteraeota* (Brewer et al., 2019), or tree root host-specific mycorrhizal fungal strains, as shown in *Eucalyptus* forests at 4 m soil depth (Robin et al., 2019). Also, it appears that microbial diversity is not necessarily lower in subsoils than in topsoils, as recently has been found on the Loess Plateau in China in soils down to 20 m depth (Liu et al., 2020).

As climate change is progressing, it is assumed that shifts in vegetation will occur throughout forest biomes (McDowell et al., 2020). In Europe, European beech (*Fagus sylvatica* L.) forest will move toward cooler sites, most likely toward the north, east, or higher elevations, and oak trees (*Quercus* spp.) will take over because they are resistant to higher temperatures and lower water availability (Hanewinkel et al., 2013). The soil microbial communities might also change in the long term, not only because climatic conditions have changed, but also because of the shift in dominant tree species. Many oak forests are less dense and shady than beech forests (often little or no understory vegetation, reminding of Gothic Cathedrals – “Buchen-Hallenwald”; Ellenberg, 1986), thus permitting more light and rainfall to reach the forest floor. In addition, the litter quality (i.e., roots, leaves, wood) of oaks might be different than that of beech, affecting soil properties in a different direction, e.g., the quality of the soil organic matter, due to a higher content of tannins and of phenolics in the bark of oaks (Torkaman and Seyam, 2009). Lastly, oak trees mostly host different ectomycorrhizal fungal species than beech trees (van der Linde et al., 2018). Overall, it is assumed that variability in soil moisture, soil temperature, and local conditions such as dominant tree species might affect the phenotypic response of the soil microbiome, with feedbacks to climate change (Jansson and Hofmockel, 2020).

Switzerland hosts some regions where European beech faces its physiological limits due to dry climatic and extreme edaphic conditions such as low soil water holding capacity (Walthert et al., 2021). Many of these dry sites are located in the Rhone Valley and the Jura Mountains. Here, European beech forests adjoin forests dominated by more heat- and drought-tolerant pubescent oak or sessile oak, and if drought and heat periods increase due to ongoing climate change, beech will be slowly displaced by oak species. European beech is known to have a suboceanic preference (Meier and Leuschner, 2008), whereas oaks have more of a submediterranean preference (Himrane et al., 2004).

The aim of this study was to investigate the vertical distribution of the soil microbiome to a depth of 2 m in drought-exposed forest ecosystems in Switzerland. We further aimed to disentangle the influence of tree species from soil depth, since the two tree genera, beech and oak, usually host different ectomycorrhizal species, and to verify the general shift from saprotrophic to ectomycorrhizal fungal dominance with increasing soil depth recently observed by Carteron et al. (2020). Finally, we aimed to investigate how the microbial community in bulk soils is related to the fine root microbial community throughout the soil profile, as pioneering studies have shown that root-associated fungi are recruited from the soil fungal community (Goldmann et al., 2016). We hypothesized that: (1) bacterial and fungal abundances decrease with soil depth, with fungal abundance decreasing more strongly than bacterial abundance; (2) microbial diversity and community structure vary with soil depth, tree type, and substrate (soil vs. roots); (3) deep soil layers select for specific and poorly known bacteria and fungi; and (4) relative abundances of saprotrophic fungi decrease more strongly with increasing soil depth than ectomycorrhizal fungi. Our study contributes to an integrated perspective on the vertical distribution of the soil microbiome in drought-exposed forests at a fine spatial scale.

## Materials and Methods

### Forest Sites

The investigated forest sites are located in the relatively warm and dry Rhone Valley (Chamoson and Saillon) and in the Jura Mountains (Neunkirch) of Switzerland with mean annual precipitation sums (MAP, 1981–2010) of 815–955 mm and mean annual temperatures (MAT, 1981–2010) of 8.5–9.4°C depending on the site (Supplementary Figure 1 and Table 1; Remund et al., 2014). Depending on water availability, the sites are covered mainly by European beech or oak species, with oak sites having slightly warmer and drier soils than European beech sites (Table 2). All forest stands have a near natural tree species composition originating from natural regeneration and are unmanaged since several decades. The stands with its admixtures of other tree species had an approximate area of 50--200 m^2^. At the beech sites, there were no admixed tree species, except at Saillon where a few scattered *Abies alba* Mill. tree individuals were recorded. At the oak sites of Chamoson and Saillon, a few scattered tree individuals of *Acer opalus* Mill. were detected next to the dominant oak *Quercus pubescens* (Willd.), and at Neunkirch, a few tree individuals of *F. sylvatica* were detected next to the dominant oak *Q. petraea* (Matt.) Liebl. The forest sites are part of the research platforms WSL Forest Soil Database and TreeNet^1^, and the forest site Neunkirch is a monitoring plot of the Long-term Forest Ecosystem Research Program LWF (ICP-Forests^2^). The soil classification followed the system of the Food and Agriculture Organization of the United Nations (IUSS Working Group WRB, 2007) (Table 1).

**TABLE 1:** Forest site, tree and soil characteristics.

	**Lat.**	**Long.**	**Topography**			**Climate**		**Trees**			**Soil type**
					
			**Elev.**	**Exp.**	**Slope**	**MAT**	**MAP**	**Age**	**Height**	**DBH**	**FAO class.**
	**N**	**E**	**m a.s.l.**		**%**	**°C**	**mm**	**Year**	**m**	**cm**	
**Beech sites:**											
Chamoson	46°12′	07°12′	880	NE	65	8.5	870	74	29	39	Eutric Cambisol
Neunkirch	47°41	08°32′	560	N	58	8.8	953	166	24	66	Rendzic Leptosol
Saillon	46°10	07°09′	890	SE	55	9.4	829	100	24	38	Calcaric Regosol
**Oak sites:**											
Chamoson	46°12′	07°12′	870	S	90	8.6	815	66	9	19	Calcaric Regosol
Neunkirch	47°40	08°32′	640	S	35	9.1	949	134	17	38	Rendzic Leptosol
Saillon	46°10	07°09′	790	SE	50	9.4	828	97	18	28	Calcaric Regosol

*Lat., latitude; Long., longitude; Elev., elevation; Exp., exposition; MAT, mean annual temperature; MAP, mean annual precipitation sum; DBH, stem diameter at breast height (mean of the three sampled trees); FAO class., classification according to IUSS Working Group WRB (2007). World Reference Base for Soil Resources 2006, first update 2007. World Soil Resources Reports No. 103. FAO, Rome.*

**TABLE 2:** Mean values of soil temperature (°C) and soil water potential (kPa) in 2015 (L2, L4, L6: *n* = 3 forest sites; L7: *n* = 2 forest sites).

	**Beech sites**				**Oak sites**				***p*-ANOVA^+^**		
**Soil layers^#^:**	**L2**	**L4**	**L6**	**L7**	**L2**	**L4**	**L6**	**L7**	**Tree**	**Depth**	**Interact.**
**Soil temperature:**											
January–March	3.8	4.9	5.9	7.2	4.4	5.4	6.7	8.2	**0.018**	**<0.001**	0.93
April–June	10.7	9.6	8.8	8.6	12.5	11.7	10.7	10.6	**<0.001**	**0.002**	0.99
July–September	15.5	14.5	13.3	12.5	17.0	16.3	14.8	14.1	**0.001**	**<0.001**	0.99
October–December	8.4	9.6	10.2	10.9	9.1	10.3	11.1	12.0	**0.011**	**<0.001**	0.94
Year	9.6	9.7	9.5	9.8	10.8	10.9	10.8	11.2	0.11	0.99	1.00
**Soil water potential:**											
January–March	−10	−57	−288	−431	−45	−387	−572	−833	0.10	0.07	0.83
April–June	−30	−21	−77	−203	−60	−93	−231	−640	**0.008**	**0.002**	0.13
July–September	−614	−497	−509	−418	−814	−778	−689	−850	0.07	0.94	0.93
October–December	−322	−579	−623	−612	−531	−874	−831	−984	0.09	0.31	0.98
Year	−244	−289	−374	−416	−362	−533	−581	−827	**0.002**	0.05	0.54

*^+^Effects of tree species, soil depth and their interaction were assessed by analysis of variance (ANOVA); significant values (*p* < 0.05) are in bold.*

*^#^Soil layers: L2: 15–25 cm, L4: 75–85 cm, L6: 140–155 cm, L7: 180–200 cm.*

### Soil and Root Sampling

Soil profiles down to 2 m depth were excavated in the center of a group of dominant trees at each study site in spring 2014. Three soil samples per soil depth were taken with 1 L steel cylinders (10.7 cm height, 11.1 cm diameter) at depths of 0–10 cm (L1), 15–25 cm (L2), 45–55 cm (L3), 75–85 cm (L4), 110–125 cm (L5), 140–155 cm (L6), and 180–200 cm (L7). However, for depths >45 cm at Neunkirch beech and oak sites and for depths >140 cm at the Chamoson oak site, the cylinders could not be used for sampling due to the very high content of large stones. Instead, bulk soil samples were excavated and the entire volume was determined by refilling the cavity with quartz sand. For soil chemical and physical analyses, approximately 1 kg of soil was sampled separately from the various depths (L1–L7). All soil samples were then transported in plastic bags to the labs and stored at 4°C until further processing.

In order to estimate fine root biomass and fine-earth densities, the soil samples of the 1 L steel cylinders were sieved with a 4 mm sieve and the roots were sorted out. The roots were then washed, separated according to their diameters (<2 mm: fine roots, 2–5 mm, >5–10 mm, >10 mm) and according to their species affiliation (beech, oak). European beech roots are recognized as being brown reddish with a rigid appearance, and oak roots as being yellowish with a rough appearance. Afterward, the soil was further sieved through a 2 mm mesh sieve.

For soil and root microbial DNA-sampling, aliquots of soils of about 5 g were put into a Ziploc^®^ bag and frozen and stored at −80°C for DNA analyses. Roots were frozen in liquid nitrogen (N_2_), freeze-dried, weighed, and stored at −20°C for DNA analyses. In deep soil layers, where steel cylinder samples could not be taken, three soil and root aliquots were sampled from the bulk soil, from which DNA samples were taken independently. Finally, the remaining soil was dried at 105°C and volumetric stone content and fine-earth density were estimated.

### Soil Chemical and Physical Analyses

The soil samples for chemical and particle size analyses were dried a 60°C and sieved through a 2 mm mesh sieve. Soil pH was determined in 0.01 M CaCl_2_. For carbon (C) and nitrogen (N) analyses, an aliquot of the dried and sieved samples was ground for 3 min using a vibrating ball mill (MM2000, Retsch GmbH, Germany) with zircon-grinding tools. Exchangeable cations (Na, K, Mg, Ca, Mn, Al, Fe) were extracted (in triplicate) in an unbuffered solution of 1 M NH_4_Cl. The element concentrations in the extracts were determined by ICP-OES (Optima 3000, Perkin–Elmer, United States). In samples with a pH (CaCl_2_) < 6.5, the exchangeable proton content was calculated as the difference between the total and the Al-induced exchangeable acidity, as determined by the KCl method (Thomas, 1982). In samples with a higher pH, the contents of exchangeable protons were assumed to be negligible. The effective cation-exchange capacity (CEC) was obtained by summing the charge equivalents of exchangeable Na, K, Mg, Ca, Mn, Al, Fe, and H. The base saturation (BS) was calculated as the ratio of the sum of exchangeable Na, K, Mg, and Ca to the CEC. Organic C (C_org_) and total N (N_tot_) were determined with a CN elemental analyzer (NC2500; CE Instruments, Italy). The organic C (C_org_) content of samples with a pH (CaCl_2_) < 6.0 was assumed to be equal to the total carbon (C_tot_) content, whereas samples with a pH > 6.0 were assumed to potentially contain carbonates and, thus, were fumigated with HCl vapor prior to the CN analysis (Walthert et al., 2010). Soil particles were fractionated into sand, silt and clay using the pipette method according to Gee and Bauder (1986). Plant-available water storage capacity (AWC), i.e., the storable amount of water between field capacity and permanent wilting point, was calculated with a pedotransfer function according to Teepe et al. (2003) for each sampled soil layer for a thickness of 10 cm considering fine-earth density and soil texture, i.e., particle size distribution.

### Measurement of Soil Temperature and Soil Water Potential

MPS-2 sensors and EM-50 data loggers (Decagon Devices, United States) for continuously recording soil temperature and soil water potential (Ψ_soil_) were installed at each study site in spring 2014. Two to three MPS-2 sensors per soil depth were embedded in the front wall of the soil pit at 20, 80, and 140 cm depth (at 180–200 cm depth only in Chamoson and Saillon). Measurements of Ψ_soil_ were temperature corrected to 22°C according to Walthert and Schleppi (2018). Soil temperature and soil water potential (Ψ_soil_) have been recorded from spring 2014 onward in hourly intervals. In order to characterize the forest sites, daily data from year 2015 were calculated and divided into four annual quarters (January–March, April–June, July–September, October–December).

### Soil and Root DNA Extraction, MiSeq Sequencing, and Bioinformatic Analyses

For DNA extraction, either frozen soil material or lyophilized fine root material, which was chopped with sterilized scissors and afterward grounded in 2 ml Eppendorf tubes containing eight sterile steel balls (2 mm diameter) on a FastPrep-24 bead beating grinder (MP Biomedicals, United States) three times for 30 s, was used. DNA was extracted using the PowerSoil DNA Isolation Kit (Qiagen, Germany). For the bead beating procedure, either 0.5 g soil or 0.02–0.1 g root material was used according to Herzog et al. (2019). DNA was quantified using the high-sensitivity Qubit assay (Thermo Fisher Scientific, Switzerland). The V3–V4 region of the bacterial small-subunit (16S) rRNA gene and the internal transcribed spacer region 2 (ITS2) of the eukaryotic (fungal groups, some groups of protists, and green algae) ribosomal operon were PCR amplified using primers previously described by Frey et al. (2016). PCR amplification was performed with 40 ng soil DNA and the GoTaq^®^ G2 HotStart Polymerase (Promega, Switzerland) in a final volume of 50 μL per samples (16S: 5 min at 95°C/36 cycles: 40 s at 94°C, 40 s at 58°C, 1 min at 72°C/10 min at 72°C; ITS-2: 5 min at 95°C/38 cycles: 40 s at 94°C, 40 s at 58°C, 1 min at 72°C/10 min at 72°C). A mixture of peptide nucleotide acid (PNA) blocker oligos (PNA Bio Inc., United States) targeted at plant mitochondrial and plastidic genomes was added in the PCR reaction mixture of root DNA to increase the fraction of bacterial sequences and reduce the PCR-bias, resulting in more accurate sequencing (Lundberg et al., 2013). Thus, the PCR mixture contained 1.25 mPNA blocker (0.25 μM) and 1.25 pPNA blocker (0.25 μM). A PNA clamping at 75°C for 10 s after denaturation at 94°C was included in the PCR conditions. Both positive and negative PCR controls were included to exclude false positives and negatives. Bacterial and fungal amplicons were sent to the Génome Québec Innovation Centre at McGill University (Montreal, QC, Canada) for barcoding using the Fluidigm Access Array technology and paired-end sequencing on the Illumina MiSeq v3 platform (Illumina Inc., United States). Raw sequences have been deposited in the NCBI Sequence Read Archive under the BioProject accession number PRJNA704912.

Quality filtering, clustering into operational taxonomic units (OTUs), and taxonomic assignment were performed as previously described in Frey et al. (2016). In brief, a customized pipeline largely based on UPARSE (Edgar, 2013; Edgar and Flyvbjerg, 2015) and implemented in USEARCH (v9.2; Edgar, 2010) was used. Filtered reads were de-replicated and singleton reads were removed prior to clustering. Sequences were clustered into OTUs at 97% sequence identity (Edgar, 2013). For taxonomic classification of the OTUs, corresponding centroid sequences were queried against selected reference databases using the naïve Bayesian classifier (Wang et al., 2007) and a minimum bootstrap support of 80%. Prokaryotic sequences were queried against the SILVA database (v132; Quast et al., 2013). Eukaryotic ITS2 sequences were first queried against a custom-made ITS2 reference database retrieved from NCBI GenBank, and sequences assigned to fungi were subsequently queried against the fungal ITS database UNITE (v8.0; Abarenkov et al., 2010). Prokaryotic centroid sequences identified as originating from organelles (chloroplast, mitochondria), as well as eukaryotic centroid sequences identified as originating from soil animals (*Metazoa*), plants (*Viridiplantae*, except green algae), or of unknown eukaryotic origin, were removed prior to data analysis.

### Quantitative PCR of Bacterial and Fungal Ribosomal Markers

Relative abundances of the bacterial 16S rRNA genes and the fungal ITS genes of soils from 0–10 cm, 45–55 cm and 140–155 cm depth were determined by quantitative PCR using an ABI7500 Fast Real-Time PCR system (Applied Biosystems, United States) and using 27f-512r (16S V1–V3) and ITS2 primers (without barcodes) and cycling conditions as for the sequencing approach (see also Frey et al., 2020). For qPCR analyses, 20 ng DNA in a total volume of 6.6 μL and 8.4 μL GoTaq^®^ qPCR Master Mix (Promega, Switzerland) containing 1.8 μM of each primer and 0.2 mg mL^–1^ of BSA were used. PNA blockers were also added in the reaction mix of the root DNA as described above. The specificity of the amplification products was confirmed by melting-curve analysis. Three standard curves per target region (correlations ≥ 0.997) were obtained using tenfold serial dilutions (10^–1^ to 10^–9^ copies) of plasmids generated from cloned targets (Frey et al., 2011). Data were converted to represent the average copy number of targets per μg DNA and per g soil.

### Data Analysis

All statistical analyses were performed using R (v4.0.2; R Core Team, 2020). We used analysis of variance (ANOVA) to test for effects of soil depth (L1: 0–10 cm, L2: 15–25 cm, L3: 45–55 cm, L4: 75–85 cm, L5: 110–125 cm, L6: 140–155 cm and L7: 180–200 cm) tree genus (beech or oak), and their interaction on univariate response variables. *Post hoc* differences between soil depths were assessed with Tukey’s HSD *post hoc* tests. All variables were tested for normality and homogeneity of variances using Shapiro–Wilk and Bartlett’s tests, respectively. In case of non-normality and/or heteroscedasticity, variables were transformed by taking either the natural logarithm or the square root of the original values.

For the analysis of bacterial and fungal α-diversities, the observed richness (number of OTUs) and Shannon diversity index were estimated based on OTU abundance matrices rarefied to the lowest number of sequences (Adamczyk et al., 2020), using the R package phyloseq (v1.32; McMurdie and Holmes, 2013). To assess the main and interactive effects of soil depth, tree species and substrate (soil or root) on α-diversities, a three-way ANOVA was performed. Pairwise differences between groups were evaluated with Tukey’s HSD *post hoc* tests.

To evaluate the differences in microbial community structures (β-diversities) between the soil samples, Bray–Curtis dissimilarities were calculated based on square root transformed relative abundances of OTUs (Hartmann et al., 2017). The effects of soil depth, tree species, substrate, as well as interactive effects, on the soil microbial β-diversities were assessed with permutational ANOVAs (PERMANOVA, number of permutations = 10^5^), using the function ‘adonis’ implemented in the R package ‘vegan’ (v2.5.6; Oksanen et al., 2019). Differences in microbial β-diversities between soils were visualized with canonical analysis of principal coordinates (CAP) ordinations (ordinate function of the R package ‘phyloseq’), constrained for the tested factors of soil depth, tree species and substrate. Microbial β-diversities were also visualized using unconstrained non-metric multidimensional scaling (NMDS) ordinations.

To evaluate the influences of the soil environmental conditions on microbial β-diversity, soil environmental variables were projected as vectors onto the NMDS ordinations using the *envfit* function in the R package ‘vegan.’ The direction of the vectors reflects the gradient of variation of the environmental variables, from the smallest (origin) to the largest (arrowhead) values. Permutation tests (10^5^ permutations) implemented with the *envfit* function were used to evaluate the statistical significance of the correlation (*R*^2^) between the projected variables and the NMDS axes.

To identify bacterial and fungal phyla and genera that were differentially distributed between topsoil (L1 and L2; 0–25 cm) and subsoil (L5 and L6; 110–155 cm) we performed DESeq2 pairwise analyses (DESeq2 package version 1.30.0; Love et al., 2014, 2020). The DESeq2 comparisons were performed at the OTU level, and the differentially abundant OTUs identified in the analyses were grouped *a posteriori* into the taxa displayed in the graphical representations. To avoid the influence of rarely occurring (and potentially erroneous) OTUs in the analyses, the DESeq2 comparisons only included OTUs for which the sum of the sequences over all soil samples belonging to the same tree species at each forest site was ≥ 10, for at least two independent combinations of forest site and tree species. For each DESeq2 comparison of topsoil vs. subsoil, the log_2_ fold change (LFC) of each OTU was calculated as the log_2_ (genera abundance of topsoil/genera abundance of subsoil). OTU abundances were normalized with the median of ratios method to account for differences in sequencing depth between samples. The statistical significance of the LFC was evaluated with Wald tests (Rüthi et al., 2020). *P* values were adjusted for multiple testing using the Benjamini–Hochberg method with a false discovery rate threshold of 5%.

The topsoil vs. subsoil DESeq2 comparisons were performed independently for (1) the subset of fine roots and (2) the subset of bulk soils, collected across all beech and oak sites at the distinct soil depths. Soil layer (topsoil or subsoil) was implemented as the main factor in the DESeq2 tests, controlling for the additional factors of site and tree species (∼ site + tree species + soil layer). DESeq2 analyses were also applied to identify differentially abundant taxa between beech and oak trees (∼ site + tree species), and soils and fine roots (∼ site + tree species + substrate). In these later cases, the DESeq2 tests included all fine roots and soil samples.

Fungal functional guilds were assigned within the six most abundant guilds, namely ectomycorrhizal fungi, arbuscular mycorrhizal fungi, endophyte, undefined saprotrophs, animal pathogens, and plant pathogens, using an open annotation tool (FUNGuild) according to Nguyen et al. (2016). Only guild assignments with ‘highly probable’ confidence rankings were accepted (Perez-Mon et al., 2020).

## Results

### Forest Site Characteristics

Analysis of soil chemical and physical parameters at the six forest sites showed that they were not dependent on the tree genus, as none of the variables revealed significances. In contrast, some of the soil parameters showed significant changes with depth (Table 3). Soil pH showed a significant increase with soil depth, whereas soil base saturation remained at a high level around 100%. Both, C_org_ and N_tot_ decreased significantly with soil depth by about tenfold, as did clay content by about fivefold. In contrast, fine-earth density, sand content, and stone content increased with soil depth, whereas C:N ratio, silt content, and the plant-available water storage capacity (AWC) remained more or less constant (Table 3).

**TABLE 3:** Mean values of soil biological, chemical and physical properties (L1–L6: *n* = 3 forest sites; L7: *n* = 2 beech sites, *n* = 1 oak site).

	**Beech sites**							**Oak sites**							***p*-ANOVA^+^**		
**Soil layers^#^:**	**L1**	**L2**	**L3**	**L4**	**L5**	**L6**	**L7**	**L1**	**L2**	**L3**	**L4**	**L5**	**L6**	**L7**	**Tree**	**Depth**	**Interaction**
**Soil biological properties:**																	
Microbial biomass (μg DNA g^–1^ soil)*	18.8	14.5	9.66	2.40	2.06	1.40	0.07	11.3	9.43	3.01	1.86	1.49	1.40	2.32	**0.050**	**<0.001**	0.37
Fine root biomass (g dm^–3^ soil)	3.29	1.78	0.69	0.37	0.21	0.37	0.37	3.90	2.65	1.03	0.50	0.20	0.41	0.44	**0.017**	**<0.001**	0.27
**Soil chemical properties:**																	
pH (CaCl_2_)	6.56	6.97	7.28	7.81	7.71	7.60	7.75	6.32	7.32	7.66	7.68	7.70	7.71	7.75	0.71	**0.002**	0.93
Base saturation (%)	98.6	99.3	99.5	99.9	100	100	100	99.5	99.9	99.9	99.9	99.9	99.9	99.9	0.33	0.26	0.87
C_org_ (%)	6.89	3.42	1.46	0.91	0.81	0.68	0.56	6.48	2.29	1.05	0.95	0.48	0.48	0.68	0.55	**<0.001**	1.00
N_tot_ (%)	0.45	0.27	0.13	0.07	0.09	0.08	0.09	0.39	0.20	0.12	0.11	0.08	0.08	0.05	0.56	**0.002**	0.99
C:N ratio	15.6	11.9	10.6	14.3	14.2	13.1	11.6	16.4	11.9	8.8	9.9	8.0	8.4	14.5	0.42	0.80	0.95
**Soil physical properties:**																	
Fine-earth density (g cm^–3^)	0.64	0.81	0.98	1.06	1.20	1.04	1.22	0.73	0.88	0.99	1.01	1.10	1.12	1.21	0.75	**<0.001**	0.79
Stones (%)	15.8	15.8	28.3	54.2	43.3	33.8	46.3	9.7	24.2	39.2	47.5	47.5	47.5	5.0	0.81	0.32	0.92
Sand (%)	17.6	16.7	18.5	31.4	32.3	45.6	45.4	22.6	36.9	34.3	40.6	42.8	44.0	36.8	0.09	**0.029**	0.63
Silt (%)	45.3	48.0	53.1	47.4	49.8	43.4	47.1	41.7	35.7	45.1	44.4	44.1	43.4	57.6	0.58	0.97	0.98
Clay (%)	37.2	35.3	28.4	21.3	18.0	14.0	7.7	35.8	27.5	20.6	15.0	13.2	12.7	5.7	0.34	**0.032**	1.00
AWC_10_ (mm)^‡^	21.6	23.1	17.3	11.2	13.3	15.5	12.9	27.0	40.0	15.1	12.6	11.9	11.8	23.2	0.40	0.14	0.83

*^+^Effects of tree genus, soil depth and their interaction were assessed by analysis of variance (ANOVA); significant values (*p* < 0.05) are in bold.*

^#^Soil layers: L1: 0–10 cm, L2: 15–25 cm, L3: 45–55 cm, L4: 75–85 cm, L5: 110–125 cm, L6: 140–155 cm, L7: 180–200 cm.

*Soil DNA content as a proxy for soil microbial biomass.

*^‡^Plant-available water storage capacity (AWC) per 10 cm soil thickness.*

Analysis of fine root variables showed, that oak sites had a slightly but significant greater fine root biomass compared with beech sites, and mainly at depths down to 1 m (Table 3). However, a strong significant decrease of the fine root biomass with increasing soil depth occurred down to 2 m for both tree species, with the strongest decrease within the first 50 cm, from around 3.5 to 0.6 g dm^3^ soil.

At the oak sites, soil temperatures of each of the yearly quarters were significantly higher than at the beech sites, by 1–2°C, whereas the yearly means within each of the soil layers were not significantly different (Table 2). The soil water potentials varied strongly within each site and within each of the yearly quarters, resulting in significantly lower water potentials for the oak sites only for the first quarter. Averaged over the whole year, however, the oak sites had significantly (1.5–2 times) lower water potentials compared with the beech sites (Table 2).

### Soil Bacteria and Fungi Abundance and Diversity

Bacteria are here defined as being bacterial and archaeal taxa. Bacterial and fungal abundances in bulk soil, as indicated by quantitative PCRs, showed no significant differences between the beech and oak sites, with a bacteria to fungi ratio of about 75–110 in the upper 50 cm of soil, and a ratio of about 400 at a depth of 150 cm (Table 4). Soil depth, in contrast, had a significant effect on fungal abundances in soils, with values decreasing up to 500 times with greater soil depth. In contrast, bacterial abundance in bulk soils tended to be higher in the subsoil. The bacteria to fungi ratio in bulk soils of both beech and oak sites increased by about five times, from around 80 to 400, from the top layer (L1) to the lowest layer (L6) (Table 4). Soil depth did not affect the bacterial or fungal abundance or their ratios in the fine root samples, whereas fungal abundance was significantly higher in beech compared with oak roots.

**TABLE 4:** Mean values of bacterial and fungal abundances (16S and ITS copy numbers) and the bacteria to fungal ratio in bulk soils and fine roots (*n* = 3 forest sites).

	**Beech sites**			**Oak sites**			***p*-ANOVA^+^**		
**Soil layers^#^:**	**L1**	**L3**	**L6**	**L1**	**L3**	**L6**	**Tree**	**Depth**	**Interaction**
**Abundance in soils:**									
Bacterial 16S (×10^8^)*	3.58	2.91	4.94	3.83	3.67	4.77	0.66	0.14	0.83
Fungal ITS (×10^6^)	6.47	4.03	1.89	5.80	3.64	1.27	0.60	**0.012**	0.99
16S:ITS ratio	75	87	384	77	109	402	0.82	**0.002**	0.99
**Abundance in roots:**									
Bacterial 16S (×10^8^)	3.17	2.35	2.62	3.59	3.70	2.60	0.10	0.20	0.26
Fungal ITS (×10^8^)	8.18	6.61	9.74	1.14	1.33	1.31	**<0.001**	0.66	0.65
16S:ITS ratio	0.47	0.45	0.38	4.45	9.56	6.31	0.06	0.79	0.79

*^+^Effects of tree genus, soil depth and their interaction were assessed by analysis of variance (ANOVA); significant values (*p* < 0.05) are in bold.*

^#^Soil layers: L1: 0–10 cm, L3: 45–55 cm, L6: 140–155 cm.

*16S and ITS copy numbers are given in μg^–1^ DNA.

After quality filtering we identified among the 234 soil samples a total of 9,256,287 bacterial sequences with an average of 39,556 ± 7,668 per sample. These sequences were distributed among 12,543 OTUs (without singletons), 95.2% of which could be classified at the phylum level (32 phyla) and 15.3% at genus level (382 genera). The predominant bacterial phyla were *Proteobacteria* (22.3%; 2385 OTUs), *Planctomycetes* (20.1%; 2235 OTUs), *Acidobacteria* (12.8%; 935 OTUs), *Verrucomicrobia* (11.2%; 684 OTUs) and *Actinobacteria* (11.1%; 957 OTUs). The most abundant genera were *Bradyrhizobium* (2.0% of relative abundance), *Streptomyces* (1.1%) and *Rhodomicrobium* (0.8%). We further identified 89 archaeal OTUs with a total of 85,662 sequences.

For fungi, sequence analysis yielded a total of 10,960,463 sequences, averaging 46,840 ± 4,711 per sample. These sequences represented a total of 3,133 fungal OTUs (without singletons), 95% of which could be classified at the phylum level (8 phyla) and 55% at genus level (467 genera). *Basidiomycota* (61.6%; 1027 OTUs), *Ascomycota* (34.7%; 1716 OTUs), and *Mortierellomycota* (3.2%, 65 OTUs) were found to be the predominant fungal phyla. The most abundant genera were *Inocybe* (29.7% of relative abundance), *Boletus* (5.3%) and *Cenococcum* (2.9%). A complete list of all bacterial and fungal OTUs, including their taxonomic assignment, the number of sequences and abundance information can be found in the Supplementary Data Sheets 3,4.

The α-diversity of bacteria showed highly significant effects of site, substrate (bulk soil vs. fine roots), tree species, and soil depth, with the one exception that the Shannon index was not significantly affected by tree species (Table 5). A similar pattern was observed for the fungal α-diversity, with the one exception that the sites had no significant effect on the Shannon index (Table 5).

**TABLE 5:** Main effects on bacterial and fungal α-diversity (Richness and Shannon index) and β-diversity (Bray–Curtis dissimilarities) of forest sites (Chamoson, Neunkirch, Saillon), soil depths (soil layers: L1: 0–10 cm, L2: 15–25 cm, L3: 45–55 cm, L4: 75–85 cm, L5: 110–125 cm, L6: 140–155 cm, L7: 180–200 cm), tree genera (beech, oaks), and substrates (bulk soil, fine roots).

		α-diversity*					
		**Richness**		**Shannon**		**β-diversity^+^**	
	***DF*^#^**	***F***	***p***	***F***	***p***	***F***	***p***
**Bacteria:**							
Site	2	51.3	**<0.001**	17.5	**<0.001**	12.8	**<0.001**
Depth	6	16.8	**<0.001**	4.9	**<0.001**	5.7	**<0.001**
Tree	1	8.4	**0.004**	0.6	0.43	7.2	**<0.001**
Substrate	1	459	**<0.001**	288	**<0.001**	77.7	**<0.001**
**Fungi:**							
Site	2	16.9	**<0.001**	1.7	0.18	8.7	**<0.001**
Depth	6	38.5	**<0.001**	4.8	**<0.001**	2.8	**<0.001**
Tree	1	8.8	**0.003**	142	**<0.001**	28.9	**<0.001**
Substrate	1	517	**<0.001**	444	**<0.001**	60.8	**<0.001**

*Effects of site, soil depth, tree genus, and substrate were assessed by analysis of variance (ANOVA).

*^+^Effects of site, soil depth, tree species and substrate were assessed by permutational multivariate analysis of variance (PERMANOVA).*

*^#^Values represent degrees of freedom *(DF*), the F-ratio (*F*), and the level of significance (*p*); significant values (*p* < 0.05) are in bold.*

**FIGURE 1:**
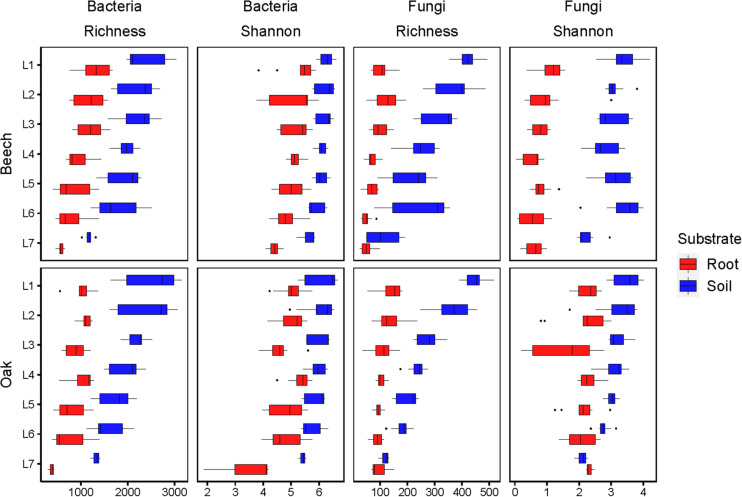
Richness and Shannon index of bacteria and fungi of soils and fine roots in the various soil layers (L1–L7) at beech and oak sites. Soil layers: L1: 0–10 cm, L2: 15–25 cm, L3: 45–55 cm, L4: 75–85 cm, L5: 110–125 cm, L6: 140–155 cm, L7: 180–200 cm.

Both, richness and the Shannon index showed a decreasing trend with increasing soil depth for both bulk soil and fine roots (Figure 1). However, the decrease in richness was much more pronounced than the decline in the Shannon index. For both bacteria and fungi, the Shannon index remained more or less constant across the soil layers L1–L6 (0–155 cm depth) and only decreased in the deepest layer L7 (180–200 cm; Figure 1). Comparing the microbial communities in the bulk soil with those of the fine roots, it is clear that both richness and the Shannon index were higher in the soil. The richness of bacteria in the soil was about 2× higher and of fungi about 4× higher compared with values for fine roots. Similarly, the Shannon index of bacteria in the bulk soil was about 1.2× and of fungi about 1.5–3× higher compared with values for fine roots (Figure 1). It is noteworthy that fungi associated with the fine roots of oaks had a distinctly higher Shannon index (about 2) than those associated with the fine roots of European beech (about 1), while fungi of the bulk soils of both forest types had about the same Shannon index (about 3) (Figure 1).

**FIGURE 2:**
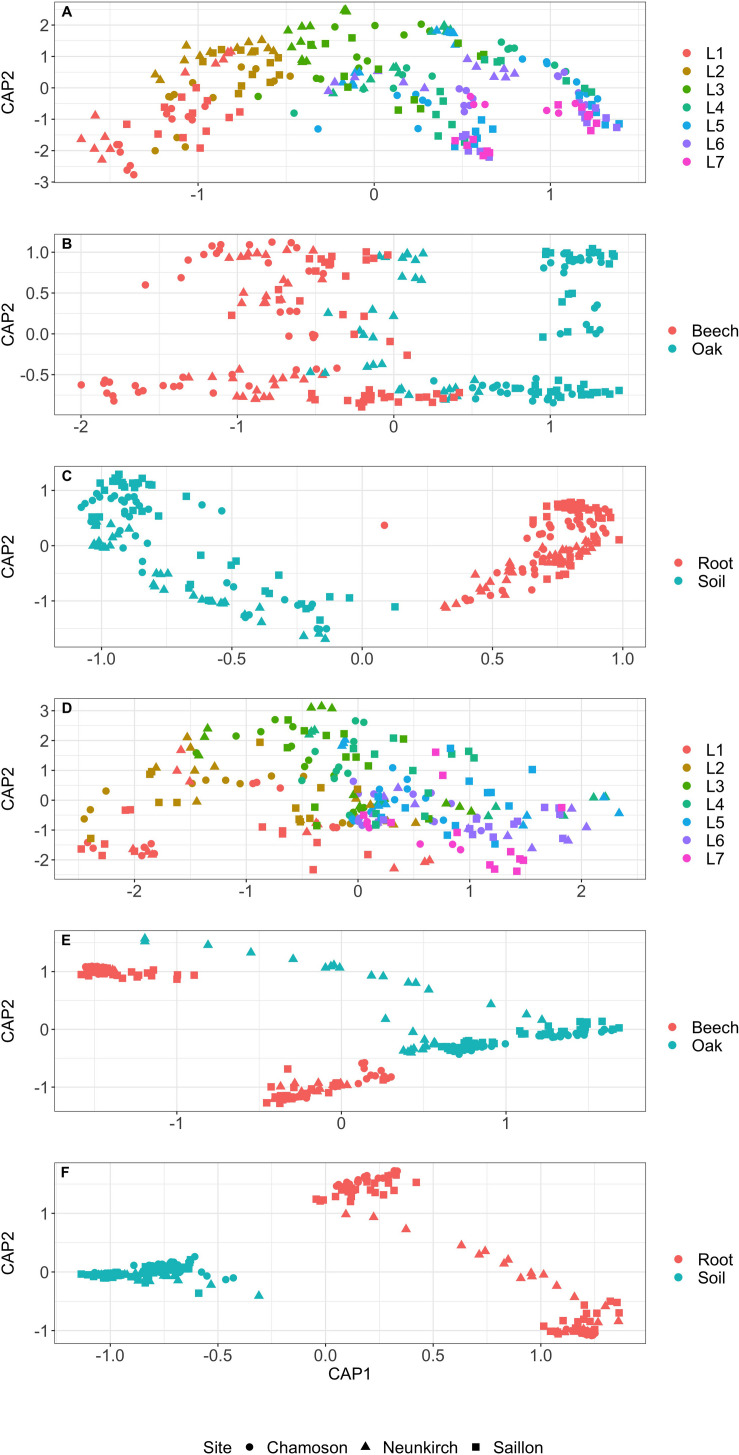
Canonical analysis of principal coordinates (CAP) based on Bray–Curtis dissimilarities for bacterial and fungal communities. **(A)** Bacterial communities in the various soil layers (L1–L7) of three forest locations. **(B)** Bacterial communities in the beech and oak sites of the three forest locations. **(C)** Bacterial communities in the soil and fine root substrates of the three forest locations. **(D)** Fungal communities in the various soil layers (L1–L7) of three forest locations. **(E)** Fungal communities in the beech and oak sites of the three forest locations. **(F)** Fungal communities in the soil and fine root substrates of the three forest locations.

PERMANOVA analyses revealed significant differences in bacterial and fungal communities (β-diversity) with site, soil depth, tree species, and substrate (Table 5). The canonical analysis of principal coordinates (CAP) based on Bray–Curtis dissimilarities for microbial communities showed that bacterial communities were significantly separated across the soil layers (L1–L7; *F* = 5.7, *p* < 0.001). However, they were less strongly dependent on tree species (beech vs. oak; *F* = 7.2, *p* < 0.001), in contrast to the substrate (bulk soil vs. fine roots; *F* = 77.7, *p* < 0.001) (Table 5 and Figures 2A–C). Fungal communities were less strongly separated across the soil layers than bacterial communities (*F* = 2.8, *p* < 0.001). However, they were strongly dependent on tree species (*F* = 28.9, *p* < 0.001) and substrate (*F* = 60.8, *p* < 0.001; Table 5 and Figures 2D–F).

**FIGURE 3:**
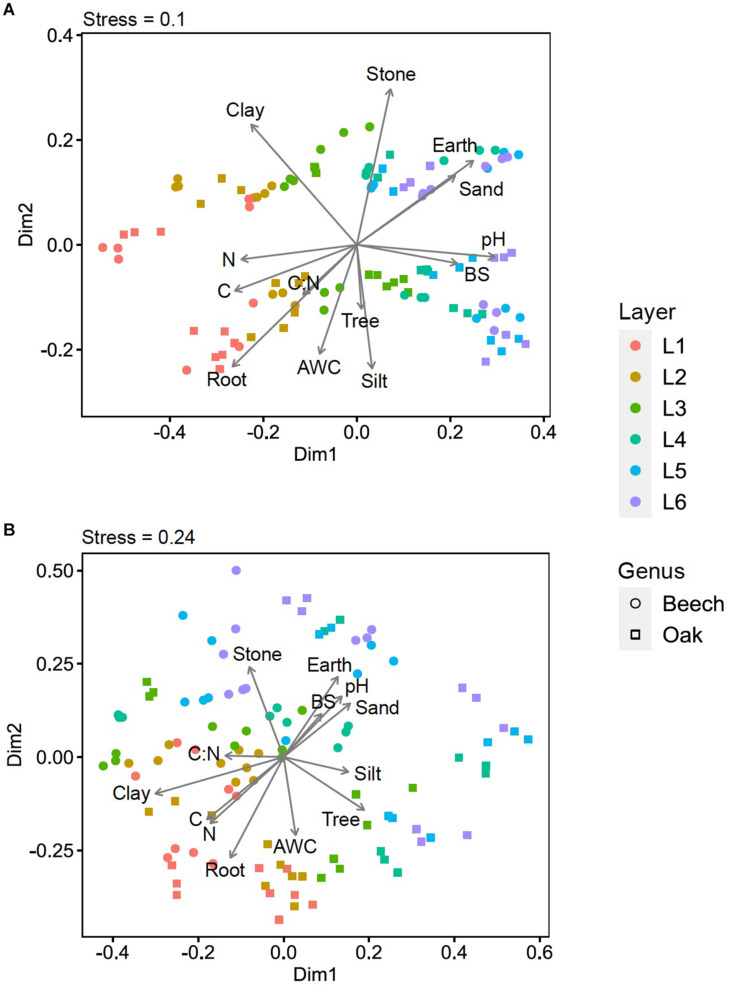
Non-metric multidimensional scaling (NMDS) ordination based on Bray–Curtis dissimilarities for **(A)** bacterial and **(B)** fungal communities in various soil layers (L1–L6) of beech and oak sites. Soil environmental variables were projected as arrow vectors onto the NMDS ordinations. Vector arrowheads show the direction of variation of the soil variables, and vector lengths reflect the strength of the correlation of the variables with the NMDS axes. AWC: plant-available water storage capacity, BS, base saturation; C, C_org_; Clay, clay content; CN, C:N ratio; Earth, fine-earth density; N, N_tot_; pH, pH (CaCl_2_); Root, fine-root biomass; Sand, sand content; Silt, silt content; Stone, stone content; Tree, tree genus. Soil layers: L1: 0–10 cm, L2: 15–25 cm, L3: 45–55 cm, L4: 75–85 cm, L5: 110–125 cm, L6: 140–155 cm (for explanations see Table 3).

The non-metric multidimensional scaling (NMDS) ordination based on Bray–Curtis dissimilarities for the microbial communities in the various soil layers (L1–L6) of beech and oak sites indicated a strong separation of the soil bacterial communities (stress = 0.1; Figure 3A). We further identified the individual soil environmental variables explaining the bacterial and fungal community structures. Correlations with the soil environmental variables revealed that fine root biomass, clay content, C_org_, and N_tot_ were highly significantly related (*R*^2^ = 0.39–0.78, *p* < 0.001) to the bacterial communities in the upper soil layers, whereas communities in the lower soil layers corresponded closely to the stone content, fine-earth density, pH, and sand content (*R*^2^ = 0.39–0.58, *p* < 0.001; Table 6). In contrast, tree species, silt content, C:N ratio, base saturation, and plant available water storage capacity (AWC) were not or only weakly related to the bacterial communities (Figure 3A). The soil fungal communities, in contrast to the bacterial communities, separated less well across the soil layers (stress = 0.24; Figure 3B). Similar to the bacterial communities, correlations with the fungal communities in the upper soil layers were high for clay content, fine root biomass, C_org_, and N_tot_ (*R*^2^ = 0.38–0.63, *p* < 0.001; Table 6). But in contrast to the bacterial communities, however, fungal communities correlated strongly with the tree genus (*R*^2^ = 0.34, *p* < 0.001). The fungal communities of the lower soil layers correlated with soil variables in a way similar to that observed for the bacterial communities (stone content, fine-earth density, pH, and sand content; *R*^2^ = 0.28–0.38, *p* < 0.001; Table 6). The fungal communities were not or only weakly related to the soil parameters silt content, C:N ratio, base saturation, and AWC (Figure 3B).

**TABLE 6:** Strength of the correlation (*R*^2^) of the soil environmental variables to microbial community dissimilarities in the various soil layers (L1–L6) of beech and oak sites calculated with a non-metric multidimensional scaling (NMDS) ordination of the Bray–Curtis dissimilarity matrix.

	**Bacteria**		**Fungi**	
	***R*^2^**	***p*^+^**	***R*^2^**	***p***
**Soil biological variables:**				
Tree genus	0.09	**0.002**	0.34	**<0.001**
Fine root biomass	0.78	**<0.001**	0.55	**<0.001**
**Soil chemical variables:**				
pH (CaCl_2_)	0.55	**<0.001**	0.29	**<0.001**
Base saturation	0.30	**<0.001**	0.13	**0.002**
C_org_	0.47	**<0.001**	0.38	**<0.001**
N_tot_	0.39	**<0.001**	0.38	**<0.001**
C:N ratio	0.14	**<0.001**	0.14	**0.042**
**Soil physical variables:**				
Fine-earth density	0.55	**<0.001**	0.38	**<0.001**
Stones	0.58	**<0.001**	0.38	**0.003**
Sand	0.39	**<0.001**	0.28	**<0.001**
Silt	0.36	0.82	0.14	0.99
Clay	0.65	**<0.001**	0.63	**<0.001**
AWC10*	0.31	**<0.001**	0.27	**0.002**

*Compare also Figure 3 for the arrow vectors and abbreviations, and Table 3 for the units.*

^+^Significant values (*p* < 0.05) are in bold.

*Plant-available water storage capacity (AWC) per 10 cm soil thickness.

### Differential Abundance of Bacterial and Fungal Taxa in Response to Soil Depth, Tree Genus and Substrate

**FIGURE 4:**
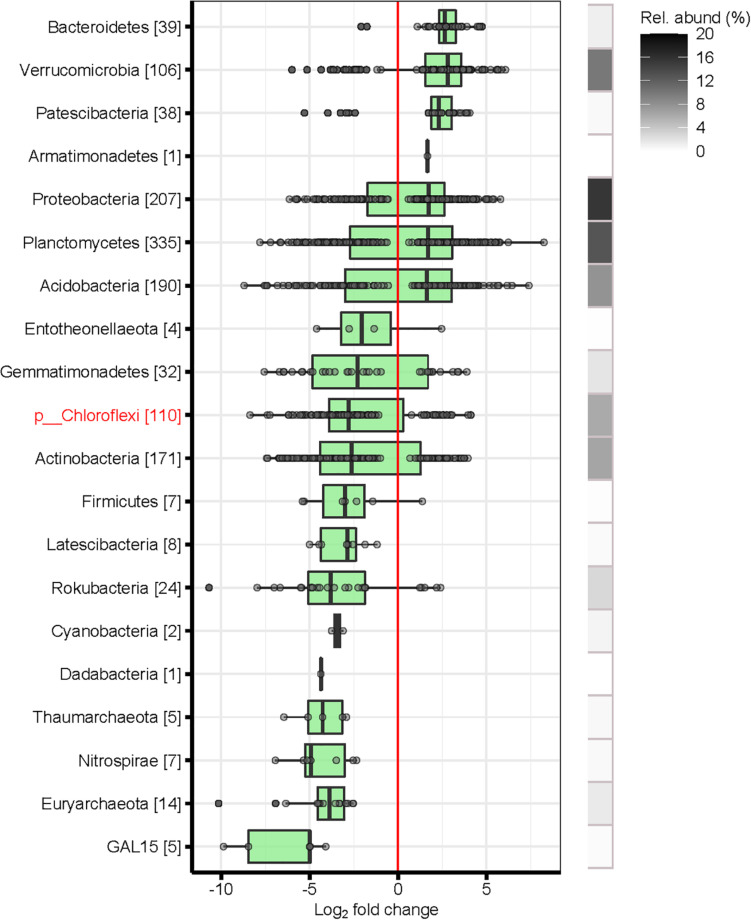
Differentially abundant bacteria phyla of topsoil (soil layers L1 and L2) vs. subsoil (soil layers L5 and L6). Shown are only significant log_2_-fold changes (LFC) of phyla based on a significance level of *p* < 0.01 after false discovery rate correction. Error bars represent standard deviation. Positive LFC values indicate a higher occurrence of phyla in the topsoil, whereas negative LFC indicate a higher occurrence in the subsoil. The number of differentially abundant OTUs comprised in each phyla are indicated in brackets. Poorly known phyla are highlighted in red. Relative abundance (%): sum of the abundance of the differentially abundant OTUs belonging to the same phyla as a percentage of the total number of sequences across the compared soils. Soil layers: L1: 0–10 cm, L2: 15–25 cm, L5: 110–125 cm, L6: 140–155 cm.

**FIGURE 5:**
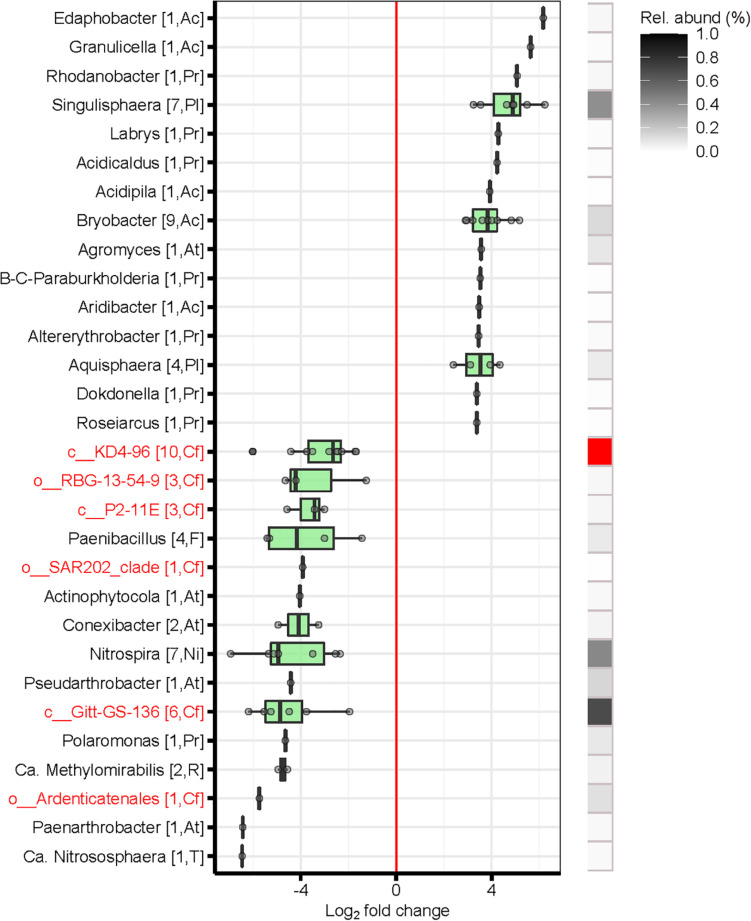
Differentially abundant bacteria genera of topsoil (soil layers L1 and L2) vs. subsoil (soil layers L5 and L6). Shown are only significant log_2_-fold changes (LFC) of genera based on a significance level of *p* < 0.01 after false discovery rate correction. Error bars represent standard deviation. Positive LFC values indicate a higher occurrence of the taxa in the topsoil, whereas negative LFC indicate a higher occurrence in the subsoil. Only 30 genera with the highest (positive) and lowest (negative) mean LFC values were represented. The number of differentially abundant OTUs comprised in each genus, and the phyla to which they belong are indicated in brackets. Poorly known taxa are highlighted in red. Relative abundance (%): sum of the abundance of the differentially abundant OTUs belonging to the same genera as a percentage of the total number of sequences across the compared soils. Relative abundance depicted in red exceeds the abundance scale. Ac, Acidobacteria; At, Actinobacteria; Cf, Chloroflexi; F, Firmicutes; Ni, Nitrospirae; Pl, Planctomycetes; Pr, Proteobacteria; R, Rokubacteria; T, Thaumarchaeota. Soil layers: L1: 0–10 cm, L2: 15–25 cm, L5: 110–125 cm, L6: 140–155 cm.

Overall, bacterial and archaeal taxa belonging to the phyla *Bacteroidetes, Verrucomicrobia, Patescibacteria, Proteobacteria, Planctomycetes* and *Acidobacteria* were significantly more abundant in topsoils than in subsoils, independently from the substrate (Figure 4). In contrast, poorly known bacterial phyla, such as *Nitrospirae*, *Chloroflexi*, *Rokubacteria*, *Gemmatimonadetes* and GAL 15, were overrepresented in the subsoils. Furthermore, archaeal phyla, such as *Thaumarchaeota* and *Euryarchaeota*, were also more abundant in subsoils than in topsoils (Figure 4). All these mentioned phyla that were overrepresented in the subsoil were also more abundant in bulk soils than in root samples (data not shown). Interestingly, we found many poorly characterized taxa (i.e., within *Chloroflexi* and *Actinobacteria*) to be more abundant in subsoils than in topsoil (Figure 5). Furthermore, the genera *Conexibacter*, *Paenarthrobacter* and *Pseudarthrobacter* (all from *Actinobacteria*), *Nitrospira* (*Nitrospirae), Paenibacillus (Firmicutes), Polaromonas (Proteobacteria)* and *Candidatus Nitrososphaera (Thaumarchaeota)* were more abundant in subsoils. In addition, several poorly known taxa from *Chloroflexi* (i.e., candidate classes Gitt-GS-136, KD4-96, and TK10; candidate orders *Ardenticatenales, SAR 202* and *RBG-13-54-9)* were overrepresented in subsoils. In contrast, the genera *Edaphobacter*, *Granulicella* and *Bryobacter* (both *Acidobacteria*) were significantly increased in topsoils relative to in subsoils. Similarly, the genera *Singulisphera* (*Planctomycetes*) and *Rhodanobacter* (*Proteobacteria*) were significantly more abundant in topsoils (Figure 5).

The abundance of *Anaerolineaceae* taxa (belonging to *Chloroflexi*) was significantly higher in bulk soil samples from beech compared with oak sites (Supplementary Figure 2). We also found that several poorly characterized bacterial taxa from *Chloroflexi* (*Anaerolineaceae*, *Ktedonobacteraceae;* candidate classes Gitt-GS-136, P2-11E and TK10; candidate orders SBR1031 and S085) were more abundant in bulk soil than in fine root samples (Supplementary Figure 3). In contrast, several root-associated bacterial taxa, such as *Pseudomonas, Rhodomicrobium* and *Streptomyces* exhibited a positive log_2_-fold change in root samples relative to bulk soil samples.

**FIGURE 6:**
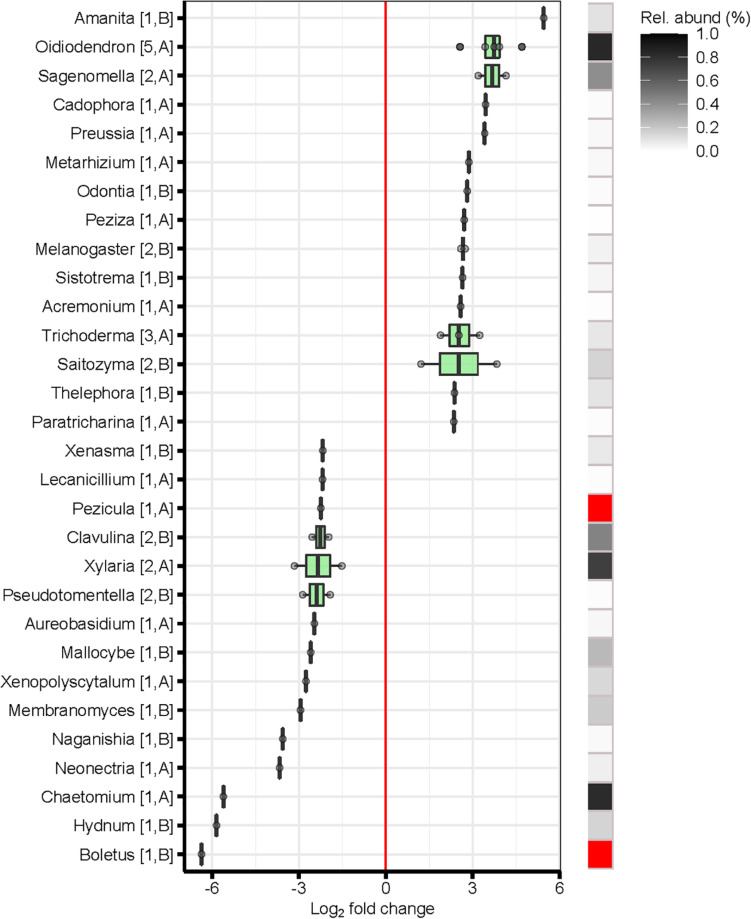
Differentially abundant fungal genera of topsoil (soil layers L1 and L2) vs. subsoil (soil layers L5 and L6). Shown are only significant log_2_-fold changes (LFC) of genera based on a significance level of *p* < 0.01 after false discovery rate correction. Error bars represent standard deviation. Positive LFC values indicate a higher occurrence of the taxa in the topsoil, whereas negative LFC indicate a higher occurrence in the subsoil. Only thirty genera with the highest (positive) and lowest (negative) mean LFC values were represented. The number of differentially abundant OTUs comprised in each genus, and the phyla to which they belong are indicated in brackets. Relative abundance (%): sum of the abundance of the differentially abundant OTUs belonging to the same genera as a percentage of the total number of sequences across the compared soils. Relative abundance depicted in red exceeds the abundance scale. A, Ascomycota; B, Basidiomycota. Soil layers: L1: 0–10 cm, L2: 15–25 cm, L5: 110–125 cm, L6: 140–155 cm.

Within the fungal kingdom, the majority of genera that exhibited either positive or negative log_2_-fold changes in abundance with soil depth (topsoil L1 and L2 vs. subsoil L5 and L6) were members of the phyla *Ascomycota* and *Basidiomycota*. The ectomycorrhizal genera *Amanita, Melanogaster, Odontia, Sistotrema*, and *Telephora* were among the fungal taxa that showed the highest positive log_2_-fold change in the topsoils. In contrast, a different group of genera of ectomycorrhizal fungi were found in the subsoils such as *Boletus, Clavulina, Hydnum, Mallocybe, Membranomyces*, and *Pseudotomentella*, and (Figure 6). The majority of fungi with the largest log_2_-fold changes in abundance with soil depth, however, were saprotrophs. Abundant saprotrophs in the topsoils were *Oidiodendron*, *Saitozyma*, and *Trichoderma*, which are all litter decomposers. In addition, *Sagenomella* was recorded, which is in contrast to the saprotrophs an animal pathogen. In the subsoils, abundant saprotrophs were members of *Xylaria*, a well-known wood decomposer (Figure 6).

Abundant ectomycorrhizal genera predominantly found in beech sites were *Hebeloma* and *Hymenogaster*, and in the oak sites *Cenococcum* and *Russula* (Supplementary Figure 4). One exception is *Glomus*, which belongs to *Glomeromycota* and forms arbuscular mycorrhizas with roots of herbs, grasses, and shrubs. Further abundant genera in beech sites were the ascomycetous yeast *Lipomyces* and the plant pathogen *Idriella. Cistella, Leohumicola*, and *Xenasma* were the most dominant saprotrophs in oak sites (Supplementary Figure 4).

Ectomycorrhizal fungi predominantly and abundantly found in the bulk soils but not in the fine roots were, e.g., *Clavulina* and *Melanogaster* (Supplementary Figure 5). In contrast, no ectomycorrhizal fungi were found exclusively in the fine roots. Differentially abundant saprotrophic fungi recorded in soils were *Geminibasidium, Saitozyma*, and *Solicoccozyma*, all basidiomycetous yeasts*; Mortierella*, a mucoromycetous saprotroph; *Oidiodendron* and *Trichoderma*, ubiquitous decomposing fungi; and *Sagenomella*, an animal pathogen (Supplementary Figure 5). When comparing the fungi from bulk soils with those from fine roots, we observed that only a few genera predominantly occurred in fine roots, and these were known decomposers of litter and wood (*Lachnum, Mycena*, *Mycenella, Pezicula, Xylaria*) (Supplementary Figure 5).

### Fungal Guilds

**FIGURE 7:**
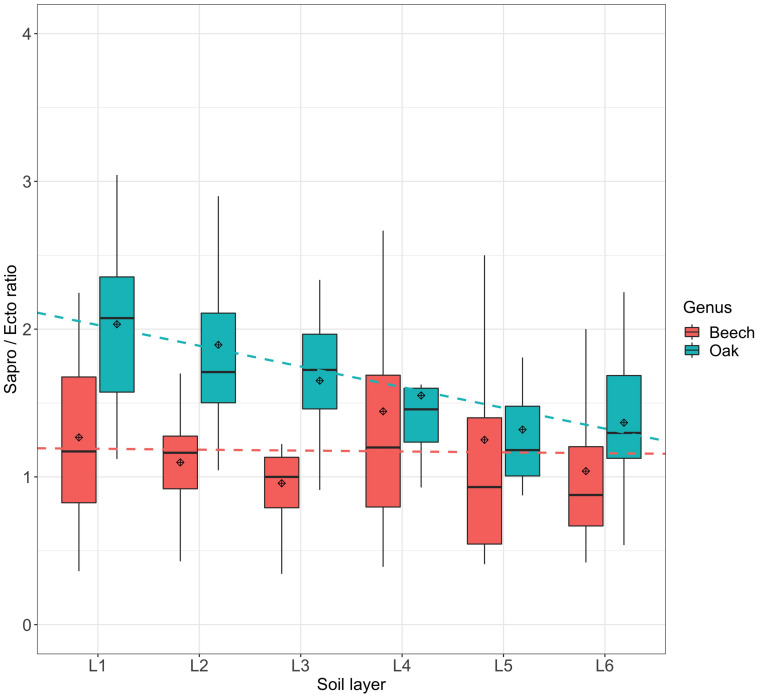
Saprotrophs to ectomycorrhizal fungi ratio of relative abundances of fungi in soils and fine roots in the various soil layers (L1–L6) at beech and oak sites. Fungal functional guilds were analyzed by FUNGuild. Dashed lines have been drawn for better visualization. Soil layers: L1: 0–10 cm, L2: 15–25 cm, L3: 45–55 cm, L4: 75–85 cm, L5: 110–125 cm, L6: 140–155 cm.

An analysis of the fungi in soils and fine roots with FUNGuild showed that in both forest types “saprotrophs” and “ectomycorrhizal fungi” dominate over “plant pathogens,” “animal pathogens,” “ericoid mycorrhizal,” and “endophyte fungi.” The latter four guilds all had a relative abundance of <10% with a “highly probable” classification (data not shown). In contrast, saprotrophs and ectomycorrhizal fungi were considerably more abundant with 27% and 41%, respectively. At beech sites, both fungal guilds were similar (37% versus 35%), whereas at oak sites saprotrophs were the dominating fungal guild (41%) compared to ectomycorrhizal fungi (27%) (data not shown). When the saprotroph to ectomycorrhizal fungi ratios of the relative abundances were compared over the soil layers, it was evident that the ratio at the oak sites decreased from around 2.1 to 1.2 with increasing soil depth, whereas at the beech sites the ratio remained more or less constant between 1.2 and 0.9 (Figure 7). Although richness and the Shannon index decreased with soil depth in both types of forest sites, the values for saprotrophs at the oak sites were much higher in the upper soil layers, and their decrease was much stronger compared with at the beech sites (data not shown). In contrast, the values for ectomycorrhizal fungi were similar at beech and oak sites, as was their decrease with increasing soil depth. A comparison of the saprotrophs with the ectomycorrhizal fungi demonstrated that these fungal guilds differed distinctly between beech and oak sites, but not between fine roots and bulk soil (Supplementary Figure 6). A complete list of the fungal guild classification can be found in the Supplementary Data Sheet 5.

## Discussion

### Life in Deep and Dry Soils

The subsurface of soils might be one of the last frontiers of undiscovered sites, also because “an increasing number of studies clearly indicate that ‘looking deeper’ is essential to increase our understanding of plant ecophysiology, community ecology and geochemical cycles” (Maeght et al., 2013). Germon et al. (2020) pointed out “the critical need to include depth and timescale of ecological processes below the soil surface in a modern conceptualization of forest ecosystems.” Only recently, it was found that particular microbial taxa are consistently more abundant in deep soils and are preferentially adapted to low-nutrient conditions, with the ability to synthesize and store carbohydrates, the potential to use carbon monoxide as a supplemental energy source, and the ability to form spores (Brewer et al., 2019). Interestingly, the few existing studies on forest ecosystems showed that the richness and diversity of soil microorganisms are not necessarily lower in deeper soil layers compared to topsoils. Moreover, specific groups of microorganisms, such as *Chloroflexi*, *Nitrospirae*, or *Euryarchaeota* emerge in the subsurface and are clearly specialized to these conditions (Brewer et al., 2019).

Extreme conditions in soils affect microbial life and biogeochemical processes (Schimel, 2018). Under conditions of periodic or permanent low water availability, which are unquestionably challenging to life, it might be expected that microbial populations would be either scarce or absent. Gremer et al. (2015) estimated that conditions become critical for soil microorganisms below a water potential of −3 MPa. To exist in dry habitats, microorganisms must be capable of surviving extended periods of low water potential. While the production of resistant spores is one option under such conditions (Manzanera, 2020), this capacity is clearly restricted to a limited range of taxa. For organisms lacking this capacity, one of the alternatives is to enter a state of anhydrobiosis (Crowe, 2014), where much of cellular metabolism is shut down and metabolic activity is restricted to processes related to cellular maintenance and repair (Manzoni and Katul, 2014; Lebre et al., 2017).

The investigated beech and oak sites, however, had maximum water potentials around −1 MPa far above values considered critical for microorganisms. However, they were critical for the trees. In the summer months of 2015, most parts of Central Europe suffered from an exceptional heat wave and dry period. The amount of precipitation in that season was among the lowest since 1901 and soil moisture was even lower than during the centennial heat wave of 2003 (Orth et al., 2016). This was also manifested specifically at our oak sites. In particular in the deep soil layers, the soil water potential reached values of −850 to −984 kPa. For young European beech trees in the oak stands, such low water potentials could be critical, as their leaves begin to drop at about −800 kPa (Walthert et al., 2021). Furthermore, such critical soil water potentials could also lead to the death of fine roots, which can then stimulate microbial activity and thereby increase soil organic matter (SOM) degradation (referred to as the rhizosphere priming effect; Adamczyk et al., 2019a).

### Soil Physicochemical Variables Affect Microbial Community Structures

Environmental conditions, such as oxygen levels, soil moisture, temperature, pH, fine-earth density, soil organic carbon content (SOC), and N content, can influence the structure of the soil microbial community (Fierer, 2017; Schimel, 2018). Therefore, changes in soil physicochemical characteristics with depth can have significant positive (e.g., SOC and N) or significant negative correlations (e.g., pH and fine-earth density) with the diversity of the microbial community. At the continental scale, soil pH has been identified as the dominant factor shaping microbial communities in surface soils (Rousk et al., 2010). In our study, however, we assumed that pH was not an important factor causing variation in microbial communities throughout the soil profiles. Our forest soil samples contained a very narrow pH range (6.6–7.8), so this variable most likely was not important in structuring the microbial communities. We expect that changes in the quality of SOC (e.g., labile SOC and resistant SOC) with soil depth have stronger effects on the community structure than the total quantity of SOC (Seuradge et al., 2017). Shifts in microbial communities between surface and subsurface soils could partially result from differences in the availability of labile SOC, leading to physiologically altered (adapted) organisms capable of utilizing more recalcitrant sources of organic carbon (Fierer et al., 2003).

A non-metric multidimensional scaling (NMDS) analysis revealed differences in microbial community structures within the soil profiles for both bacteria and fungi, although more distinctly for bacteria. The major factor that was related to microbial community structure was soil depth together with fine root biomass and C_org_, N_tot_, and clay content. This result is consistent with the observations of Fierer et al. (2007) that the availability and quality of nutrient resources appear to have a major influence on microbial community composition in soil profiles. Our topsoils contained significantly larger amounts of fine roots, C_org_, N_tot_, and clay than subsoils, both at beech and oak forest sites. This result also indicates that fine roots strongly influence the amounts of C_org_ and N_tot_ in soils (see also Rasse et al., 2005). Clay minerals, on the other hand, are associated with C_org_ because they bind strongly together and can persist for thousands of years (Rümpel and Kögel-Knabner, 2011). The decrease in C_org_ and N_tot_ content in soils as the depth increased was consistent with findings from previous studies (Du et al., 2017, 2020; Jiao et al., 2018; Feng et al., 2019). Organic C content typically declines with soil depth because of reduced nutrient input from plant litter and root exudates (Fierer et al., 2003). The decrease in microbial biomass, measured in our study as DNA content, with increasing soil depth was also consistent with results from previous studies (Eilers et al., 2012; Hu et al., 2019; Zhang et al., 2019). At our forest sites, DNA content decreased drastically from the surface soil to a depth of about 0.5 m, and then the value kept relatively steady at greater depths.

### Microbial Abundance and Diversity in Deep Soils

Our understanding of soil microbial ecology remains particularly limited with respect to changes in microbial communities with depth in particular in forests (Pickles and Pither, 2014). Nonetheless, many important biogeochemical processes, such as C_org_ and N cycling, are carried out differently throughout the soil profile by distinct microbial assemblages (Eilers et al., 2012). Here, soil microbiomes followed a vertical distribution in terms of their abundance, diversity and community structure. We found consistent decreases in bacterial alpha-diversity with increasing depth, corroborating findings from previous studies on forests (Fierer et al., 2003; Seuradge et al., 2017; Deng et al., 2018; Jiao et al., 2018; Brewer et al., 2019; Feng et al., 2019). Our results go further, showing that fungal diversity in both beech and oak soil profiles also decreased with increasing depth. This trend of decreasing fungal richness was much stronger than the pattern observed for bacteria. Bacterial and fungal community structure also showed a clear vertical stratification with depth, in agreement with previous studies (Deng et al., 2018; Tang et al., 2018; Feng et al., 2019; Hao et al., 2021). In particular, bacterial communities in the topsoils (0–25 cm soil depth) were clearly separated from those in the deeper layers.

The vertical differences in these microbiomes can be attributed in large part to the decrease in availability of various resources with increasing soil depth (Wu et al., 2020). If oxygen levels also decrease with soil depth, then this could also have contributed to the observed microbiome differences, since different microorganisms prefer different oxygen conditions – archaeal taxa are mainly anaerobic, while bacteria and fungi are mainly aerobic (Fontaine et al., 2007; Haroon et al., 2013).

### Shifts in Bacterial Taxa With Soil Depth

Our results demonstrate that the composition of the soil microbiome shows stratum specificity across the 2 m o soil profile. At a coarse taxonomic resolution we identified several bacterial and archaeal phyla that consistently increased in abundance with increasing soil depth, as measured by DESeq2 analyses across the entire data set: *Nitrospirae, Chloroflexi*, *Firmicutes*, *Rokubacteria*, *Thaumarchaeota, Gemmatimonadetes, Euryarchaeota*, and GAL15. Their presence in subsoils may indicate an adaptation of these members to the low-nutrient environments characteristic of bulk soils.

Members of *Chloroflexi* and *Nitrospirae* have previously been found to increase in abundance with increasing soil depth in individual soil profiles (Will et al., 2010; Brewer et al., 2019; Feng et al., 2019), while candidate phylum GAL15 has been shown to be abundant in sedimentary subsoils (Lin et al., 2012; Gocke et al., 2017; Brewer et al., 2019; Yan et al., 2019; Liu et al., 2020; Hao et al., 2021), floodplain soils (Steger et al., 2019), and forest subsoils (Feng et al., 2019). Members of these phyla are likely oligotrophic taxa adapted to surviving under the resource-limited conditions found in deeper layers. In the study of Yan et al. (2019) the candidate phylum GAL15 was sensitive to variation in most of the soil properties and was significantly negatively correlated with SOM, N_tot_, available K, and available P in soil profiles down to 3 m in agricultural land planted with legumes. In addition to GAL15, the subsoil also contained a greater relative abundance of *Rokubacteria* than in topsoils, which is in accordance with our findings.

Several members of *Chloroflexi* were shown to be enriched in the subsoil. In particular, we found several uncultivable bacteria from the candidate classes Gitt-GS-136 and SBR1031 within *Chloroflexi*. Studying uncultivable bacteria provides an opportunity to explore poorly characterized species of bacteria, thus providing insight into their diversity and also helping to understand their role in deep soil layers. *Chloroflexi* is a diverse phylum including autotrophic, heterotrophic and mixotrophic taxa (Hanada et al., 2002; Bennett et al., 2020). Thus, different physiological strategies might be responsible for them coping with the harsh environments in subsoils. *Chloroflexi* have also been found to be highly abundant in C-poor alpine environments at high elevations (Rime et al., 2016; Adamczyk et al., 2019b).

Two other phyla, *Gemmatimonadetes* and *Nitrospirae*, were also found in greater relative abundance in the subsoil. Previous research has highlighted that *Gemmatimonadetes* members are strongly adapted to low soil moisture conditions (DeBruyn et al., 2011; Rime et al., 2015). Furthermore, members from the *Nitrospirae* phylum have been shown to prefer non-rhizosphere environments (Dunbar et al., 2002; Steger et al., 2019). Will et al. (2010) hypothesized that heterotrophic microorganisms that are associated with roots suppress the growth of autotrophic *Nitrospirae*. Considering the general decrease in C_org_ and N_tot_ with depth observed in our study, the greater abundance of members of the *Nitrospirae* phylum in deeper soil may be due to a selection advantage that dark-adapted chemolithoautotrophic members have in subsoil systems (Will et al., 2010).

Although archaeal taxa were relatively rare in all soil layers in our study, some individual members of *Archaea* were abundant. *Thaumarchaeota* was the dominant taxon of archaeal organisms in our forest soils. Interestingly, *Thaumarchaeota* was more abundant in subsoils, which is in contrast findings from other studies (Mikkonen et al., 2014) but supports a study by Liu et al. (2020). *Thaumarchaeota* are generally negatively correlated with C_org_ (Liu et al., 2020) and are able to compete with various groups of bacteria in soils (Hansel et al., 2008). *Thaumarchaeota* have been shown to have ammonia oxidation (Treusch et al., 2005) and C sequestration activities (Koenneke et al., 2014). The relative abundance of *Euryarchaeota* increased significantly with increasing depth in our forest soils, which could be attributed to their preference for anaerobic environment. Notably, members of *Euryarchaeota* produce methane (Bodelier and Steenbergh, 2014), but also oxidize methane, fix N, and reduce nitrates (Cabello et al., 2004). With respect to archaeal phyla, which were overrepresented in the subsoils in our study, we conclude that they play a major role in soil nutrient cycling in deep layers.

Overall, bacteria are known to be involved in various soil processes and global biogeochemical cycling, such as organic matter degradation, and N cycling. The presence of chemolithotrophic prokaryotes (i.e., *Chloroflexi*, *Nitrospirae*, GAL15, and *Archaea*) in deep soils suggests that the microbial community is supported by chemolithotrophy, with limited utilization of organic carbon. Besides chemolithotrophy in the deep-soil community, metabolic cooperation via syntrophy between archaeal and bacterial groups plays a critical role in the survival of the whole community under oligotrophic conditions. Our study sheds light on the vertical spatial variation of the forest soil microbiomes at a fine scale.

In contrast, the relative abundance of *Verrucomicrobia, Bacteroidetes, Planctomycetes* and *Proteobacteria* was significantly higher in topsoils (0–25 cm) than in subsoils (110–155 cm) in our study, which is consistent with previous studies (Will et al., 2010; Feng et al., 2019). *Verrucomicrobia* may prefer a micro-aerobic environment rather than aerobic and extreme anoxic environments (Eilers et al., 2012). The decreased abundances of *Proteobacteria* and *Bacteroidetes* with increasing soil depth could be attributed to the general copiotrophic properties and responses to labile sources of C_org_ at shallow depths (Fierer et al., 2007). Soil organic carbon may affect the relative microbial abundance of *Planctomycetes* across the soil profile. Liu et al. (2020) reported that the abundance of *Planctomycetes* was significantly correlated with soil organic carbon, indicating that copiotrophic conditions benefit the growth of this phylum.

### Shifts in Fungal Taxa With Soil Depth

Previous studies on microbial communities in deep soil layers have been focused predominantly on bacteria (e.g., Li et al., 2014; Gocke et al., 2017; Liu et al., 2020). In studies where fungal taxa were considered, soil samples were mainly taken from topsoils (e.g., 0–10 cm; Egidi et al., 2019). One exception is the study by Jiao et al. (2018) investigating bacterial, archaeal, and fungal communities in soils down to 3 m on reforestation sites with *Robinia pseudoacacia* L. on the Loess Plateau in China. The authors observed only a slight decrease of the Shannon index from around 5.0 to about 4.4 in the deepest soil layers. As *R. pseudoacacia* is not considered an ectomycorrhizal forming tree species, dominant fungal taxa at the deep layers were ascomycetous fungi, such as *Cladosporium, Chaetomium* and *Trichoderma*, all three of which are saprotrophic *taxa, Gibberella* and *Stagonosporopsis*, both plant pathogens, or *Metarhizium*, an animal pathogen (Jiao et al., 2018). The additional occurring and frequent genus *Lysurus* was an exception, as it belongs to the saprotrophic *Basidiomycota* and forms stinkhorns as fruiting bodies. Recent studies exploring soils below 1 m depth have been dedicated to searching in particular for ectomycorrhizal fungi associated with deep-rooting trees. Robin et al. (2019) recorded *Pisolithus* and *Scleroderma* species in association with roots of *Eucalyptus grandis* W. Hill ex Maiden throughout the soil profiles down to 4 m in Brazil.

In our study, we also identified a *Pisolithus* species, *Pisolithus arrhizus* (Scop.: Pers.) Rauschert, at depths of 0.5–1.5 m in the soil samples of an oak site. However, this species was only rarely recorded, and thus not recognized by our differential abundance analysis. More commonly, we recorded in deep soil layers the ectomycorrhizal genera *Boletus* and *Hydnum*, and to a lesser extent *Clavulina*, *Mallocybe, Membranomyces*, and *Pseudotementella. Boletus luridus* Schaeff. (Syn. *Suillellus luridus* (Schaeff.) Murrill) was the only member of *Boletus*, but was commonly found in deep soil layers in beech and oak sites, and in both, soil and fine root samples. In addition three *Hydnum* taxa were recorded, but only *Hydnum vesterholtii* Olariaga, Grebenc, Salcedo, Martin, occurred on an abundant scale. *Hydnum vesterholtii* was recently newly described in the French Pyrenees under *Fagus sylvatica* and *Abies alba* on calcareous soils (Olariaga et al., 2017). More records are available from the Toscana region in Italy, the Huesca region in Spain, and Andorra under *Fagus, Quercus, Castanea, Corylus, Abies*, and *Pinus* (Olariaga et al., 2017). To our knowledge, our observations with *B. luridus and H. versterholtii* are the first to suggest that these fungal taxa most likely colonize tree roots in deep soil layers.

The most abundant group of fungi with the largest log_2_-fold changes in deep soil layers, however, were the saprotrophic litter and wood decomposers *Chaetomium, Pezicula*, and *Xylaria*. *Chaetomium* taxa are typically ascomycetous saprotrophs and have the ability to degrade cellulose (Dreyfuss, 1976). More than 400 species have been described, some of which can cause human infection (Wang et al., 2014). In our subsoils, an unknown *Chaetomium* taxa dominates mainly in the oak sites with a few records in the beech sites. *Pezicula*, another ascomycetous fungal genera, is predominantly reported from temperate regions, where they occur as saprotrophs on recently dead branches and twigs. However, they have also been frequently isolated from living twigs and roots without showing symptoms of disease, thus exhibiting an endophytic lifestyle (Chen et al., 2016). The dominant taxa in our study was *Pezicula brunnea* (Sigler) P. R. Johnst., which occurred throughout the soil profiles in beech and oak sites and in bulk soil and fine roots, but with a strong preference for deep soil layers. This fungus was originally isolated from roots of an ericaceous plant from Canada and described as *Cryptosporiopsis brunnea* Sigler (Sigler et al., 2005). The genus *Xylaria* is a well-known group of fungi that decompose wood. As a special feature, these fungi produce the black pigment melanin that makes their hyphae more resistant against stressors such as drought (Tudor et al., 2013). Of the several *Xylaria* taxa found, two unknown taxa were most abundant in deep soil layers, one predominantly in beech sites, the other in oak sites.

Noteworthy in deeper soil layers is the record of an additional basidomycetous genus, *Naganishia*. The most abundant species was *Naganishia albida* (Saito) X.Z. Liu (Syn. *Cryptococcus albidus*), an extremophilic black-melanized yeast, which can be found frequently in soils of extreme environments, such as Antarctica or the Atacama Desert in Chile (Schmidt et al., 2017). *Naganishia albida* is also known as a human pathogen, but it is not harmful to plants. In addition, we recorded in deep soil layers an unknown tree pathogen of the genus *Neonectria*. Many species of *Neonectria* can induce bark cankers, such as *N. major* (Castlebury et al., 2006), which we also recorded in our forest sites but mainly in the topsoils.

The ectomycorrhizal genera *Amanita, Melanogaster*, and *Telephora* were among the fungal taxa that showed the largest positive log_2_-fold change to colonize the topsoils. But overall, the most common ectomycorrhizal genera of the two forest types were *Inocybe* with >30 taxa, *Cortinarius* with >25 taxa, and *Russula* with >15 taxa. Among them, *Amanita pantherina* and *Cortinarius subtilis* occurred exclusively in the topsoils of both forest types.

### Effects of Tree Genus and Substrate Type on Microbial Communities

Our findings demonstrate that tree genera affected both the bacterial and fungal community structure. However, the fungal community structure (*F* = 28.9; *p* < 0.001) showed a much stronger dependence on tree species than the bacterial community structure (*F* = 7.2; *p* < 0.001). Thus, our results confirm the hypothesis that fungal communities are more affected by the predominant forest vegetation than the bacterial communities, which supports the findings of other studies (e.g., Urbanová et al., 2015). These authors explained the lower dependence of bacterial communities from tree roots as resulting from the unicellularity and size of most bacteria. Bacteria are expected to be exposed to the conditions of their immediate microhabitats, which are often the size of a single soil pore and which have very specific conditions that differ from the average properties of their environmental matrix (Vos et al., 2013). Due to their size, most of these microniches are likely separated from the direct influence of plant roots and may be the reason why bacterial communities in soils under different trees are affected to lesser extent than fungi (Urbanová et al., 2015).

Fierer et al. (2003) suggested that the influence of plants on soil microbial community composition is typically limited to the 20-cm topsoil layer. In contrast, we observed that the effect of tree species on fungal community could reach a depth of 2 m. At least part of the effect of tree species on soil fungi could be due to indirect effects mediated through specific understory vegetation and litter chemistry (Urbanová et al., 2015). Tree species induce a specific signature in terms of litter quality, and litter is the primary source of soil C_org_ and N, meaning that it determines the substrate quality for the growth of soil microorganisms (Baldrian, 2019). For example, oak bark has a higher tannin and total phenolic content compared to beech bark, with values of about 22% for oak and 15% for beech (Torkaman and Seyam, 2009). For the ectomycorrhizal communities we also observed a distinct difference between the two tree species, which is not surprising, as a recent study throughout Europe also showed tree specificity (van der Linde et al., 2018). Urbanová et al. (2015) concluded, that ectomycorrhizal fungi are more strongly associated with tree composition than with soil properties, in contrast to saprotrophic fungi, which are more strongly correlated with soil properties. Schappe et al. (2020) found similar relationships in tropical rainforests in Panama.

In our study, we also found that the ratio of saprotrophs to ectomycorrhizal fungi remained more or less constant at the beech sites with increasing soil depth, at values of about 1, whereas at the oak sites the ratio decreased from about 2 to 1. Therefore, our fourth hypothesis, that the relative abundances of saprotrophic fungi decrease more strongly with soil depth than ectomycorrhizal fungi, was confirmed for oak but not for beech sites. This observation at the oak sites supports the so-called Gadgil effect, which describes the increase of saprotrophic fungi when ectomycorrhizal fungi are excluded by trenching (Gadgil and Gadgil, 1971, 1975). In other words, “competition between these two fungal guilds is hypothesized to lead to suppression of decomposition rates” (Fernandez and Kennedy, 2016). At our oak sites, the ectomycorrhizal fungi occurred in a relatively lower abundance, so that competition between these two guilds would be reduced, which would promote the saprotrophs and, consequently, the decomposition of litter. The need for more saprotrophs could be explained by the fact that oak litter has a higher recalcitrance than beech litter, due to a high phenolic content. For the beech sites, however, a suppression of decomposition due to the high relative abundance of ectomycorrhizal fungi was not observed. Nevertheless, the soil C_org_ content of the two forest site types was similar.

It is likely that the microbial communities of fine roots are similar to those in the surrounding soil, as the soil represents the inoculum pool that can potentially colonize the roots (Goldmann et al., 2016). However, only a portion of this soil inoculum pool is actively associated with the roots. In German beech-dominated forests, Goldmann et al. (2016) found that root-associated fungi are recruited from the soil fungal community. Similarly, in Scots pine forests in France and Spain, Rincón et al. (2015) found that the total fungal richness in soils largely doubled that of root tips, and that more than 93% of the taxa in root tips were also found in bulk soil. These authors pointed out that bulk soil is a good proxy for estimating ectomycorrhizal fungal richness. Our study leads to a similar conclusion, that the community structure in the fine roots is, for both bacteria and fungi, well represented in the soils.

## Conclusion

Microorganisms in deeper soil layers are considered to play important roles in soil development and biogeochemical cycling, given their proximity to parent material, their potential for nutrient and carbon storage, and their lower turnover rates than surface soil microbiota. Our results from 2 m soil profiles demonstrate that the richness, abundance, and composition of the soil microbiome display layer specificity and distinct differences across strata. Soil depth is an important factor affecting microbial diversity and structure, and the gradient in the soil environment caused by soil depth, reflecting shifts in availability of resources and local chemical conditions, may be the most important factor in the vertical distribution characteristics of the microbial taxa. In particular, for bacteria we found several poorly characterized taxa in the subsoils with unknown function. Given the presence of chemolithotrophic bacteria at depth, we conclude that the microbial community is supported by chemolithotrophy with limited utilization of organic carbon. We also observed, to our knowledge for the first time, that *Boletus luridus and Hydnum versterholtii* colonize tree roots, most likely in deep soil layers. Further research is needed to evaluate their functional roles in these often-dry subsoils. Our results provide important baseline data for how microorganisms are distributed within soils, demonstrating the potential for increased phylogenetic novelty in relation to soil depth. Additional investigations surveying deeper soils may reveal novel organisms that are responsible for keystone functions in subsurface soils (Fierer, 2017; Baldrian, 2019). By focusing on deep soil layers in particular, future studies could provide insight into unique and potentially significant processes affecting drought-prone forest ecosystems.

## Data Availability Statement

The datasets presented in this study can be found in online repositories. The names of the repository/repositories and accession number(s) can be found in the article/Supplementary Material.

## Author Contributions

BF and IB designed the microbial study. LW established and maintained the six forest sites within the TreeNet network. LW, RK, and IB provided soil, fine root, and climatic data. BS performed genetic analyses in the lab. BF, CP-M, AD, and IB performed statistical analyses. BF and IB wrote the main parts of the manuscript. All authors contributed to the final version of the manuscript.

## Conflict of Interest

The authors declare that the research was conducted in the absence of any commercial or financial relationships that could be construed as a potential conflict of interest.

## Footnotes

1 www.treenet.info

2 www.icp-forests.net

## Supplementary Material

Supplementary Data Sheet 1 Supplementary Tables 1–4.

Supplementary Data Sheet 2 Supplementary Figures 1–6.

Supplementary Data Sheet 3 Bacterial OTU, Tax table.

Supplementary Data Sheet 4 Fungal OTU, Tax table.

Supplementary Data Sheet 5 Fungal guild classification, Table.
