# Supplementary material for: Deep Soil Layers of Drought-Exposed Forests Harbor Poorly Known Bacterial and Fungal Communities
Source: Front Microbiol. 2021 May 7;12:674160. doi: 10.3389/fmicb.2021.674160 (PMC8137989; doi:10.3389/fmicb.2021.674160)
Supplement: Supplementary Data Sheet 1 — Supplementary Tables 1–4. [file Data_Sheet_1.PDF]

| **Table S1.** Biological, chemical and physical properties of bulk soils | | | | | | | | | | | | | | | | |
|---|---|---|---|---|---|---|---|---|---|---|---|---|---|---|---|---|
| | | | | | | | | | | | | | | | | |
| **Soil Layer (Nr)** | **Soil Layer (cm)** | **Tree** | **Site** | **DNA soil (ng DNA g-1)** | **Fine root biomass (g dm-3 soil)** | **pH (CaCl2)** | **BS (%)** | **C-org (%)** | **N-tot (%)** | **C:N ratio** | **Fine earth density (g cm-3 soil)** | **Stones (%)** | **Sand (%)** | **Silt (%)** | **Clay (%)** | **AWC_10 cm soil (mm)** |
| L1 | 000-010 | Beech | Chamoson | 21237 | 3.18 | 5.08 | 96.1 | 4.17 | 0.31 | 13.60 | 0.72 | 5.0 | 12.8 | 54.2 | 33.0 | 21.5 |
| L2 | 015-025 | Beech | Chamoson | 11196 | 1.71 | 5.96 | 98.2 | 1.08 | 0.15 | 7.13 | 0.98 | 5.0 | 21.7 | 49.5 | 28.9 | 27.0 |
| L3 | 045-055 | Beech | Chamoson | 4185 | 0.34 | 6.48 | 98.6 | 0.64 | 0.11 | 5.74 | 1.04 | 5.0 | 18.4 | 56.0 | 25.6 | 20.8 |
| L4 | 075-085 | Beech | Chamoson | 571 | 0.04 | 7.84 | 99.9 | 0.25 | 0.07 | 3.40 | 0.99 | 62.5 | 59.5 | 28.9 | 11.7 | 9.5 |
| L5 | 110-125 | Beech | Chamoson | 136 | 0.02 | 7.56 | 100.0 | 0.29 | 0.14 | 2.02 | 1.26 | 62.5 | 55.6 | 34.2 | 10.2 | 7.8 |
| L6 | 140-155 | Beech | Chamoson | 78 | 0.03 | 7.56 | 100.0 | 0.29 | 0.14 | 2.02 | 1.26 | 62.5 | 55.6 | 34.2 | 10.2 | 7.8 |
| L7 | 180-200 | Beech | Chamoson | 31 | 0.13 | 7.76 | 100.0 | 0.31 | 0.14 | 2.30 | 1.27 | 87.5 | 55.2 | 34.7 | 10.2 | 2.6 |
| L1 | 000-010 | Beech | Neunkirch | 19432 | 3.06 | 7.34 | 99.9 | 10.50 | 0.73 | 14.40 | 0.57 | 37.5 | 23.0 | 21.8 | 55.3 | 18.8 |
| L2 | 015-025 | Beech | Neunkirch | 23903 | 1.14 | 7.49 | 100.0 | 7.03 | 0.53 | 13.30 | 0.67 | 37.5 | 11.7 | 32.1 | 56.2 | 18.8 |
| L3 | 045-055 | Beech | Neunkirch | 20814 | 0.72 | 7.73 | 100.0 | 2.68 | 0.19 | 14.20 | 0.92 | 62.5 | 15.0 | 44.3 | 40.8 | 10.7 |
| L4 | 075-085 | Beech | Neunkirch | 4506 | 0.59 | 7.90 | 100.0 | 1.24 | 0.04 | 27.60 | 1.21 | 62.5 | 12.0 | 53.8 | 34.3 | 8.8 |
| L5 | 110-125 | Beech | Neunkirch | 3746 | 0.23 | 7.90 | 100.0 | 1.24 | 0.04 | 27.60 | 1.21 | 62.5 | 12.0 | 53.8 | 34.3 | 8.8 |
| L6 | 140-155 | Beech | Neunkirch | 1779 | 0.23 | 7.50 | 99.9 | 0.92 | 0.06 | 16.40 | 0.69 | . | . | 36.7 | 26.7 | . |
| L7 | 180-200 | Beech | Neunkirch | . | . | . | . | . | . | . | . | . | . | . | . | . |
| L1 | 000-010 | Beech | Saillon | 15722 | 3.62 | 7.24 | 99.7 | 5.98 | 0.32 | 18.90 | 0.64 | 5.0 | 17.0 | 59.9 | 23.2 | 24.4 |
| L2 | 015-025 | Beech | Saillon | 8499 | 2.48 | 7.45 | 99.8 | 2.14 | 0.14 | 15.40 | 0.78 | 5.0 | 16.8 | 62.4 | 20.9 | 23.5 |
| L3 | 045-055 | Beech | Saillon | 3976 | 1.00 | 7.62 | 99.9 | 1.07 | 0.09 | 11.80 | 0.98 | 17.5 | 22.1 | 59.1 | 18.8 | 20.4 |
| L4 | 075-085 | Beech | Saillon | 2127 | 0.48 | 7.70 | 99.9 | 1.24 | 0.10 | 12.00 | 0.98 | 37.5 | 22.8 | 59.4 | 17.9 | 15.4 |
| L5 | 110-125 | Beech | Saillon | 2293 | 0.38 | 7.67 | 100.0 | 0.91 | 0.07 | 12.90 | 1.12 | 5.0 | 29.2 | 61.4 | 9.4 | 23.2 |
| L6 | 140-155 | Beech | Saillon | 2343 | 0.86 | 7.73 | 100.0 | 0.81 | 0.04 | 20.90 | 1.17 | 5.0 | 35.5 | 59.4 | 5.2 | 23.2 |
| L7 | 180-200 | Beech | Saillon | 1379 | 0.60 | 7.73 | 100.0 | 0.81 | 0.04 | 20.90 | 1.17 | 5.0 | 35.5 | 59.4 | 5.2 | 23.2 |
| L1 | 000-010 | Oak | Chamoson | 9533 | 3.62 | 6.81 | 99.6 | 8.68 | 0.53 | 16.50 | 0.61 | 5.0 | 26.6 | 45.1 | 28.4 | 28.5 |
| L2 | 015-025 | Oak | Chamoson | 11601 | 2.89 | 7.50 | 99.8 | 2.47 | 0.24 | 10.20 | 0.93 | 5.0 | 40.5 | 38.8 | 20.8 | 27.8 |
| L3 | 045-055 | Oak | Chamoson | 3886 | 1.16 | 7.79 | 99.9 | 0.79 | 0.15 | 5.19 | 1.04 | 17.5 | 37.4 | 43.7 | 18.9 | 16.3 |
| L4 | 075-085 | Oak | Chamoson | 1193 | 0.45 | 7.79 | 99.9 | 0.79 | 0.15 | 5.19 | 1.04 | 17.5 | 37.4 | 43.7 | 18.9 | 16.3 |
| L5 | 110-125 | Oak | Chamoson | 910 | 0.34 | 7.71 | 99.9 | 0.54 | 0.14 | 3.75 | 1.09 | 37.5 | 44.9 | 40.7 | 14.5 | 12.4 |
| L6 | 140-155 | Oak | Chamoson | 776 | 0.59 | 7.71 | 99.9 | 0.54 | 0.14 | 3.75 | 1.09 | 37.5 | 44.9 | 40.7 | 14.5 | 12.3 |
| L7 | 180-200 | Oak | Chamoson | . | . | . | . | . | . | . | . | . | . | . | . | . |
| L1 | 000-010 | Oak | Neunkirch | 12160 | 4.35 | 5.56 | 99.1 | 7.87 | 0.47 | 16.70 | 0.88 | 17.5 | 20.6 | 16.8 | 62.7 | 24.8 |
| L2 | 015-025 | Oak | Neunkirch | 7666 | 2.07 | 7.11 | 99.9 | 3.41 | 0.27 | 12.60 | 0.90 | 62.5 | 47.3 | 4.5 | 48.2 | 9.2 |
| L3 | 045-055 | Oak | Neunkirch | 2842 | 0.95 | 7.49 | 100.0 | 1.47 | 0.14 | 10.90 | 0.97 | 62.5 | 32.4 | 33.3 | 34.4 | 10.7 |
| L4 | 075-085 | Oak | Neunkirch | 1613 | 0.44 | 7.55 | 100.0 | 1.19 | 0.09 | 14.10 | 1.03 | 87.5 | 51.3 | 31.0 | 17.7 | 3.0 |
| L5 | 110-125 | Oak | Neunkirch | 1158 | 0.00 | 7.66 | 100.0 | 0.35 | 0.04 | 9.72 | 1.11 | 87.5 | 49.0 | 32.4 | 18.7 | 3.1 |
| L6 | 140-155 | Oak | Neunkirch | 872 | 0.29 | 7.66 | 100.0 | 0.35 | 0.04 | 9.72 | 1.11 | 87.5 | 49.0 | 32.4 | 18.7 | 3.1 |
| L7 | 180-200 | Oak | Neunkirch | . | . | . | . | . | . | . | . | . | . | . | . | . |
| L1 | 000-010 | Oak | Saillon | 12333 | 3.73 | 6.58 | 99.7 | 2.89 | 0.18 | 16.00 | 0.71 | 5.0 | 20.6 | 63.2 | 16.2 | 27.8 |
| L2 | 015-025 | Oak | Saillon | 9018 | 2.99 | 7.36 | 99.9 | 0.99 | 0.08 | 12.90 | 0.80 | 5.0 | 22.9 | 63.6 | 13.6 | 83.2 |
| L3 | 045-055 | Oak | Saillon | 2305 | 1.00 | 7.69 | 99.9 | 0.88 | 0.09 | 10.30 | 0.97 | 37.5 | 33.2 | 58.4 | 8.4 | 18.3 |
| L4 | 075-085 | Oak | Saillon | 2769 | 0.61 | 7.69 | 99.9 | 0.88 | 0.09 | 10.30 | 0.97 | 37.5 | 33.2 | 58.4 | 8.4 | 18.3 |
| L5 | 110-125 | Oak | Saillon | 2390 | 0.25 | 7.72 | 99.9 | 0.56 | 0.05 | 10.60 | 1.09 | 17.5 | 34.5 | 59.1 | 6.5 | 20.1 |
| L6 | 140-155 | Oak | Saillon | 2540 | 0.35 | 7.76 | 99.9 | 0.55 | 0.05 | 11.70 | 1.15 | 17.5 | 38.2 | 57.1 | 4.8 | 20.1 |
| L7 | 180-200 | Oak | Saillon | 2321 | 0.44 | 7.75 | 99.9 | 0.68 | 0.05 | 14.50 | 1.21 | 5.0 | 36.8 | 57.6 | 5.7 | 23.2 |

**Table S2.** Mean soil temperature and soil water potentials in 2015

| Soil layer (nr) | Soil layer (cm) | Tree | Site | Annual quarter | Temp (C) | WatPot (kPa) | | Soil layer (nr) | Soil layer (cm) | Tree | Site | Annual quarter | Temp (C) | WatPot (kPa) |
|---|---|---|---|---|---|---|---|---|---|---|---|---|---|---|
| L2 | 015-025 | Beech | Chamoson | Jan-Mar | 3.64 | -9.9 | | L2 | 015-025 | Oak | Chamoson | Jan-Mar | 5.37 | -111.9 |
| L4 | 075-085 | Beech | Chamoson | Jan-Mar | 4.68 | -16.2 | | L4 | 075-085 | Oak | Chamoson | Jan-Mar | 6.22 | -968.3 |
| L6 | 140-155 | Beech | Chamoson | Jan-Mar | 5.48 | -40.8 | | L6 | 140-155 | Oak | Chamoson | Jan-Mar | 7.49 | -842.4 |
| L7 | 180-200 | Beech | Chamoson | Jan-Mar | 6.95 | -84.8 | | L7 | 180-200 | Oak | Chamoson | Jan-Mar | 8.15 | -794.8 |
| L2 | 015-025 | Beech | Neunkirch | Jan-Mar | 3.29 | -8.8 | | L2 | 015-025 | Oak | Neunkirch | Jan-Mar | 4.01 | -9.7 |
| L4 | 075-085 | Beech | Neunkirch | Jan-Mar | 4.63 | -9.1 | | L4 | 075-085 | Oak | Neunkirch | Jan-Mar | 4.92 | -10.1 |
| L6 | 140-155 | Beech | Neunkirch | Jan-Mar | 5.54 | -10.8 | | L6 | 140-155 | Oak | Neunkirch | Jan-Mar | 5.97 | -9.2 |
| L7 | 180-200 | Beech | Neunkirch | Jan-Mar | . | . | | L7 | 180-200 | Oak | Neunkirch | Jan-Mar | . | . |
| L2 | 015-025 | Beech | Saillon | Jan-Mar | 4.46 | -11.7 | | L2 | 015-025 | Oak | Saillon | Jan-Mar | 3.95 | -13.1 |
| L4 | 075-085 | Beech | Saillon | Jan-Mar | 5.46 | -147.0 | | L4 | 075-085 | Oak | Saillon | Jan-Mar | 5.11 | -183.4 |
| L6 | 140-155 | Beech | Saillon | Jan-Mar | 6.62 | -813.8 | | L6 | 140-155 | Oak | Saillon | Jan-Mar | 6.53 | -865.0 |
| L7 | 180-200 | Beech | Saillon | Jan-Mar | 7.46 | -777.0 | | L7 | 180-200 | Oak | Saillon | Jan-Mar | 8.27 | -870.9 |
| L2 | 015-025 | Beech | Chamoson | Apr-Jun | 10.69 | -25.4 | | L2 | 015-025 | Oak | Chamoson | Apr-Jun | 13.12 | -113.9 |
| L4 | 075-085 | Beech | Chamoson | Apr-Jun | 9.73 | -14.4 | | L4 | 075-085 | Oak | Chamoson | Apr-Jun | 12.47 | -240.9 |
| L6 | 140-155 | Beech | Chamoson | Apr-Jun | 8.63 | -14.0 | | L6 | 140-155 | Oak | Chamoson | Apr-Jun | 11.13 | -462.9 |
| L7 | 180-200 | Beech | Chamoson | Apr-Jun | 8.22 | -19.6 | | L7 | 180-200 | Oak | Chamoson | Apr-Jun | 10.57 | -695.0 |
| L2 | 015-025 | Beech | Neunkirch | Apr-Jun | 10.70 | -25.3 | | L2 | 015-025 | Oak | Neunkirch | Apr-Jun | 11.39 | -20.4 |
| L4 | 075-085 | Beech | Neunkirch | Apr-Jun | 9.25 | -18.0 | | L4 | 075-085 | Oak | Neunkirch | Apr-Jun | 10.31 | -10.7 |
| L6 | 140-155 | Beech | Neunkirch | Apr-Jun | 8.44 | -17.3 | | L6 | 140-155 | Oak | Neunkirch | Apr-Jun | 9.41 | -9.4 |
| L7 | 180-200 | Beech | Neunkirch | Apr-Jun | . | . | | L7 | 180-200 | Oak | Neunkirch | Apr-Jun | . | . |
| L2 | 015-025 | Beech | Saillon | Apr-Jun | 10.73 | -39.5 | | L2 | 015-025 | Oak | Saillon | Apr-Jun | 13.08 | -44.6 |
| L4 | 075-085 | Beech | Saillon | Apr-Jun | 9.92 | -30.8 | | L4 | 075-085 | Oak | Saillon | Apr-Jun | 12.30 | -27.6 |
| L6 | 140-155 | Beech | Saillon | Apr-Jun | 9.24 | -199.9 | | L6 | 140-155 | Oak | Saillon | Apr-Jun | 11.50 | -221.7 |
| L7 | 180-200 | Beech | Saillon | Apr-Jun | 8.96 | -387.0 | | L7 | 180-200 | Oak | Saillon | Apr-Jun | 10.58 | -585.4 |
| L2 | 015-025 | Beech | Chamoson | Jul_Sep | 15.27 | -518.7 | | L2 | 015-025 | Oak | Chamoson | Jul_Sep | 17.50 | -1174.5 |
| L4 | 075-085 | Beech | Chamoson | Jul_Sep | 14.34 | -345.6 | | L4 | 075-085 | Oak | Chamoson | Jul_Sep | 16.81 | -1149.8 |
| L6 | 140-155 | Beech | Chamoson | Jul_Sep | 12.97 | -121.1 | | L6 | 140-155 | Oak | Chamoson | Jul_Sep | 14.95 | -835.3 |
| L7 | 180-200 | Beech | Chamoson | Jul_Sep | 11.91 | -104.7 | | L7 | 180-200 | Oak | Chamoson | Jul_Sep | 14.02 | -763.2 |
| L2 | 015-025 | Beech | Neunkirch | Jul_Sep | 15.69 | -608.2 | | L2 | 015-025 | Oak | Neunkirch | Jul_Sep | 15.71 | -380.9 |
| L4 | 075-085 | Beech | Neunkirch | Jul_Sep | 14.20 | -467.8 | | L4 | 075-085 | Oak | Neunkirch | Jul_Sep | 14.69 | -291.8 |
| L6 | 140-155 | Beech | Neunkirch | Jul_Sep | 13.06 | -651.8 | | L6 | 140-155 | Oak | Neunkirch | Jul_Sep | 13.53 | -270.2 |
| L7 | 180-200 | Beech | Neunkirch | Jul_Sep | . | . | | L7 | 180-200 | Oak | Neunkirch | Jul_Sep | . | . |
| L2 | 015-025 | Beech | Saillon | Jul_Sep | 15.68 | -713.7 | | L2 | 015-025 | Oak | Saillon | Jul_Sep | 17.71 | -885.2 |
| L4 | 075-085 | Beech | Saillon | Jul_Sep | 15.03 | -678.9 | | L4 | 075-085 | Oak | Saillon | Jul_Sep | 17.29 | -892.9 |
| L6 | 140-155 | Beech | Saillon | Jul_Sep | 13.88 | -753.8 | | L6 | 140-155 | Oak | Saillon | Jul_Sep | 16.04 | -961.7 |
| L7 | 180-200 | Beech | Saillon | Jul_Sep | 13.04 | -731.7 | | L7 | 180-200 | Oak | Saillon | Jul_Sep | 14.25 | -936.6 |
| L2 | 015-025 | Beech | Chamoson | Oct-Dec | 8.42 | -214.2 | | L2 | 015-025 | Oak | Chamoson | Oct-Dec | 10.14 | -861.1 |
| L4 | 075-085 | Beech | Chamoson | Oct-Dec | 9.41 | -501.3 | | L4 | 075-085 | Oak | Chamoson | Oct-Dec | 11.20 | -1255.6 |
| L6 | 140-155 | Beech | Chamoson | Oct-Dec | 9.73 | -237.8 | | L6 | 140-155 | Oak | Chamoson | Oct-Dec | 11.78 | -994.7 |
| L7 | 180-200 | Beech | Chamoson | Oct-Dec | 10.43 | -237.2 | | L7 | 180-200 | Oak | Chamoson | Oct-Dec | 12.00 | -895.9 |
| L2 | 015-025 | Beech | Neunkirch | Oct-Dec | 8.04 | -406.8 | | L2 | 015-025 | Oak | Neunkirch | Oct-Dec | 8.64 | -286.2 |
| L4 | 075-085 | Beech | Neunkirch | Oct-Dec | 9.29 | -441.9 | | L4 | 075-085 | Oak | Neunkirch | Oct-Dec | 9.49 | -303.9 |
| L6 | 140-155 | Beech | Neunkirch | Oct-Dec | 9.84 | -622.8 | | L6 | 140-155 | Oak | Neunkirch | Oct-Dec | 10.26 | -408.7 |
| L7 | 180-200 | Beech | Neunkirch | Oct-Dec | . | . | | L7 | 180-200 | Oak | Neunkirch | Oct-Dec | . | . |
| L2 | 015-025 | Beech | Saillon | Oct-Dec | 8.88 | -346.0 | | L2 | 015-025 | Oak | Saillon | Oct-Dec | 8.55 | -444.7 |
| L4 | 075-085 | Beech | Saillon | Oct-Dec | 10.22 | -793.0 | | L4 | 075-085 | Oak | Saillon | Oct-Dec | 10.31 | -1062.0 |
| L6 | 140-155 | Beech | Saillon | Oct-Dec | 10.96 | -1008.7 | | L6 | 140-155 | Oak | Saillon | Oct-Dec | 11.40 | -1089.7 |
| L7 | 180-200 | Beech | Saillon | Oct-Dec | 11.34 | -987.8 | | L7 | 180-200 | Oak | Saillon | Oct-Dec | 12.02 | -1072.3 |

**Table S3.** 16S and ITS copy numbers of bulk soils and fine roots

| Soil layer (nr) | Soil layer (cm) | Tree | Site | Soil 16S Copies per µg DNA | Soil ITS Copies per µg DNA | Soil 16S:ITS ratio | Root 16S Copies per µg DNA | Root ITS Copies per µg DNA | Root 16S:ITS ratio |
|---|---|---|---|---|---|---|---|---|---|
| L1 | 000-010 | Beech | Chamoson | 322231922 | 8845996 | 38.9 | 295172403 | 1205688368 | 0.25 |
| L3 | 045-055 | Beech | Chamoson | 253038198 | 3545228 | 76.5 | 118567394 | 792652464 | 0.13 |
| L6 | 140-155 | Beech | Chamoson | 732268949 | 1134939 | 642.0 | 158038255 | 1533454497 | 0.11 |
| L1 | 000-010 | Beech | Neunkirch | 195834305 | 1778004 | 117.2 | 357835234 | 758501050 | 0.50 |
| L3 | 045-055 | Beech | Neunkirch | 287561442 | 2237135 | 129.3 | 315018815 | 376218616 | 0.89 |
| L6 | 140-155 | Beech | Neunkirch | 395508767 | 981436 | 407.4 | 289497612 | 469481558 | 0.65 |
| L1 | 000-010 | Beech | Saillon | 555544878 | 8782813 | 69.1 | 297517527 | 490361904 | 0.68 |
| L3 | 045-055 | Beech | Saillon | 333644538 | 6301930 | 54.2 | 270406821 | 813325734 | 0.33 |
| L6 | 140-155 | Beech | Saillon | 353303781 | 3546938 | 101.6 | 338355946 | 920064738 | 0.37 |
| L1 | 000-010 | Oak | Chamoson | 415563160 | 7933687 | 57.3 | 373283484 | 57493737 | 8.23 |
| L3 | 045-055 | Oak | Chamoson | 470234741 | 4151455 | 117.4 | 359539581 | 17492667 | 24.52 |
| L6 | 140-155 | Oak | Chamoson | 356773245 | 1082965 | 330.5 | 346873398 | 21509863 | 16.11 |
| L1 | 000-010 | Oak | Neunkirch | 307163852 | 3969459 | 84.2 | 353681517 | 163837247 | 2.18 |
| L3 | 045-055 | Oak | Neunkirch | 287676617 | 4371496 | 66.5 | 332324058 | 207371090 | 1.61 |
| L6 | 140-155 | Oak | Neunkirch | 505985314 | 1749052 | 289.7 | 188808112 | 251099336 | 0.77 |
| L1 | 000-010 | Oak | Saillon | 425537489 | 5509243 | 89.4 | 348960302 | 119427614 | 2.93 |
| L3 | 045-055 | Oak | Saillon | 343668224 | 2394033 | 143.3 | 418099778 | 172893611 | 2.56 |
| L6 | 140-155 | Oak | Saillon | 569281216 | 972638 | 586.4 | 243911084 | 119709052 | 2.04 |

**Table S4.** Bacterial and fungal richness and Shannon index of bulk soils and fine roots

| Soil layer (nr) | Soil layer (cm) | Tree | Site | Bact_Richn_Soil | Bact_Shann_Soil | Bact_Richn_Roots | Bact_Shann_Roots | Fung_Richn_Soil | Fung_Shann_Soil | Fung_Richn_Roots | Fung_Shann_Roots |
|---|---|---|---|---|---|---|---|---|---|---|---|
| L1 | 000-010 | Beech | Chamoson | 2021 | 5.98 | 897 | 4.55 | 467 | 3.94 | 84.0 | 1.11 |
| L2 | 015-025 | Beech | Chamoson | 1717 | 5.80 | 804 | 4.01 | 280 | 2.96 | 69.0 | 0.41 |
| L3 | 045-055 | Beech | Chamoson | 1828 | 5.85 | 900 | 4.57 | 232 | 2.76 | 66.7 | 0.46 |
| L4 | 075-085 | Beech | Chamoson | 1836 | 5.92 | 775 | 5.04 | 199 | 2.40 | 52.0 | 0.15 |
| L5 | 110-125 | Beech | Chamoson | 1450 | 5.81 | 620 | 4.61 | 127 | 2.96 | 37.0 | 0.80 |
| L6 | 140-155 | Beech | Chamoson | 1308 | 5.70 | 530 | 4.40 | 101 | 2.70 | 32.0 | 0.10 |
| L7 | 180-200 | Beech | Chamoson | 1179 | 5.83 | 591 | 4.48 | 51 | 2.45 | 32.3 | 0.33 |
| L1 | 000-010 | Beech | Neunkirch | 2119 | 6.32 | 1549 | 5.67 | 410 | 2.99 | 97.3 | 0.96 |
| L2 | 015-025 | Beech | Neunkirch | 2307 | 6.37 | 1528 | 5.63 | 434 | 3.00 | 119.7 | 0.91 |
| L3 | 045-055 | Beech | Neunkirch | 2380 | 6.33 | 1505 | 5.62 | 351 | 2.70 | 105.0 | 0.87 |
| L4 | 075-085 | Beech | Neunkirch | 1992 | 6.25 | 1242 | 5.37 | 351 | 2.70 | 82.0 | 0.76 |
| L5 | 110-125 | Beech | Neunkirch | 2206 | 6.36 | 1273 | 5.60 | 291 | 3.12 | 88.7 | 0.78 |
| L6 | 140-155 | Beech | Neunkirch | 2341 | 6.26 | 1116 | 5.31 | 335 | 3.87 | 57.0 | 0.57 |
| L7 | 180-200 | Beech | Neunkirch | . | . | . | . | . | . | . | . |
| L1 | 000-010 | Beech | Saillon | 2901 | 6.52 | 1424 | 5.61 | 398 | 3.30 | 139.0 | 1.33 |
| L2 | 015-025 | Beech | Saillon | 2621 | 6.54 | 1230 | 5.70 | 400 | 3.40 | 176.0 | 1.82 |
| L3 | 045-055 | Beech | Saillon | 2588 | 6.45 | 1191 | 5.38 | 367 | 3.63 | 115.7 | 0.98 |
| L4 | 075-085 | Beech | Saillon | 2113 | 6.09 | 859 | 5.05 | 304 | 3.35 | 78.3 | 0.70 |
| L5 | 110-125 | Beech | Saillon | 2158 | 6.04 | 622 | 4.77 | 243 | 3.16 | 71.7 | 0.87 |
| L6 | 140-155 | Beech | Saillon | 1672 | 5.67 | 660 | 4.79 | 320 | 3.53 | 63.7 | 1.04 |
| L7 | 180-200 | Beech | Saillon | 1167 | 5.47 | 556 | 4.33 | 172 | 2.07 | 72.7 | 0.87 |
| L1 | 000-010 | Oak | Chamoson | 2738 | 6.49 | 844 | 4.87 | 466 | 3.66 | 146.7 | 2.63 |
| L2 | 015-025 | Oak | Chamoson | 2634 | 6.40 | 1008 | 5.04 | 428 | 3.72 | 99.3 | 2.37 |
| L3 | 045-055 | Oak | Chamoson | 2396 | 6.32 | 706 | 4.45 | 322 | 3.12 | 56.7 | 0.40 |
| L4 | 075-085 | Oak | Chamoson | 2220 | 6.25 | 1078 | 5.31 | 248 | 3.37 | 97.0 | 2.06 |
| L5 | 110-125 | Oak | Chamoson | 1804 | 6.09 | 762 | 4.85 | 223 | 3.20 | 83.7 | 2.26 |
| L6 | 140-155 | Oak | Chamoson | 1377 | 5.55 | 579 | 4.62 | 185 | 2.96 | 76.3 | 1.98 |
| L7 | 180-200 | Oak | Chamoson | . | . | . | . | . | . | . | . |
| L1 | 000-010 | Oak | Neunkirch | 1856 | 5.41 | 1146 | 4.96 | 427 | 3.20 | 109.7 | 1.96 |
| L2 | 015-025 | Oak | Neunkirch | 1686 | 5.35 | 1194 | 4.74 | 290 | 3.33 | 125.0 | 1.33 |
| L3 | 045-055 | Oak | Neunkirch | 1935 | 5.55 | 1114 | 5.06 | 234 | 2.97 | 121.7 | 1.89 |
| L4 | 075-085 | Oak | Neunkirch | 2165 | 5.58 | 1196 | 5.65 | 261 | 2.57 | 106.7 | 2.31 |
| L5 | 110-125 | Oak | Neunkirch | 2084 | 6.18 | 1167 | 5.48 | 233 | 2.98 | 100.0 | 1.57 |
| L6 | 140-155 | Oak | Neunkirch | 1984 | 6.18 | 1210 | 5.52 | 208 | 2.75 | 103.3 | 1.77 |
| L7 | 180-200 | Oak | Neunkirch | . | . | . | . | . | . | . | . |
| L1 | 000-010 | Oak | Saillon | 3046 | 6.57 | 1101 | 5.26 | 450 | 3.46 | 160.7 | 2.17 |
| L2 | 015-025 | Oak | Saillon | 2893 | 6.40 | 1127 | 5.33 | 366 | 2.58 | 192.7 | 2.73 |
| L3 | 045-055 | Oak | Saillon | 2213 | 6.34 | 791 | 4.18 | 277 | 3.61 | 139.0 | 2.52 |
| L4 | 075-085 | Oak | Saillon | 1555 | 5.95 | 841 | 4.96 | 200 | 3.28 | 107.0 | 2.51 |
| L5 | 110-125 | Oak | Saillon | 1297 | 5.42 | 476 | 4.27 | 152 | 2.91 | 96.0 | 2.47 |
| L6 | 140-155 | Oak | Saillon | 1313 | 5.40 | 434 | 4.21 | 143 | 2.56 | 83.0 | 2.51 |
| L7 | 180-200 | Oak | Saillon | 1322 | 5.42 | 373 | 3.39 | 117 | 2.09 | 99.7 | 2.33 |
