# Supplementary material for: Deep Soil Layers of Drought-Exposed Forests Harbor Poorly Known Bacterial and Fungal Communities
Source: Front Microbiol. 2021 May 7;12:674160. doi: 10.3389/fmicb.2021.674160 (PMC8137989; doi:10.3389/fmicb.2021.674160)
Supplement: Supplementary Data Sheet 2 — Supplementary Figures 1–6. [file Data_Sheet_2.PDF]

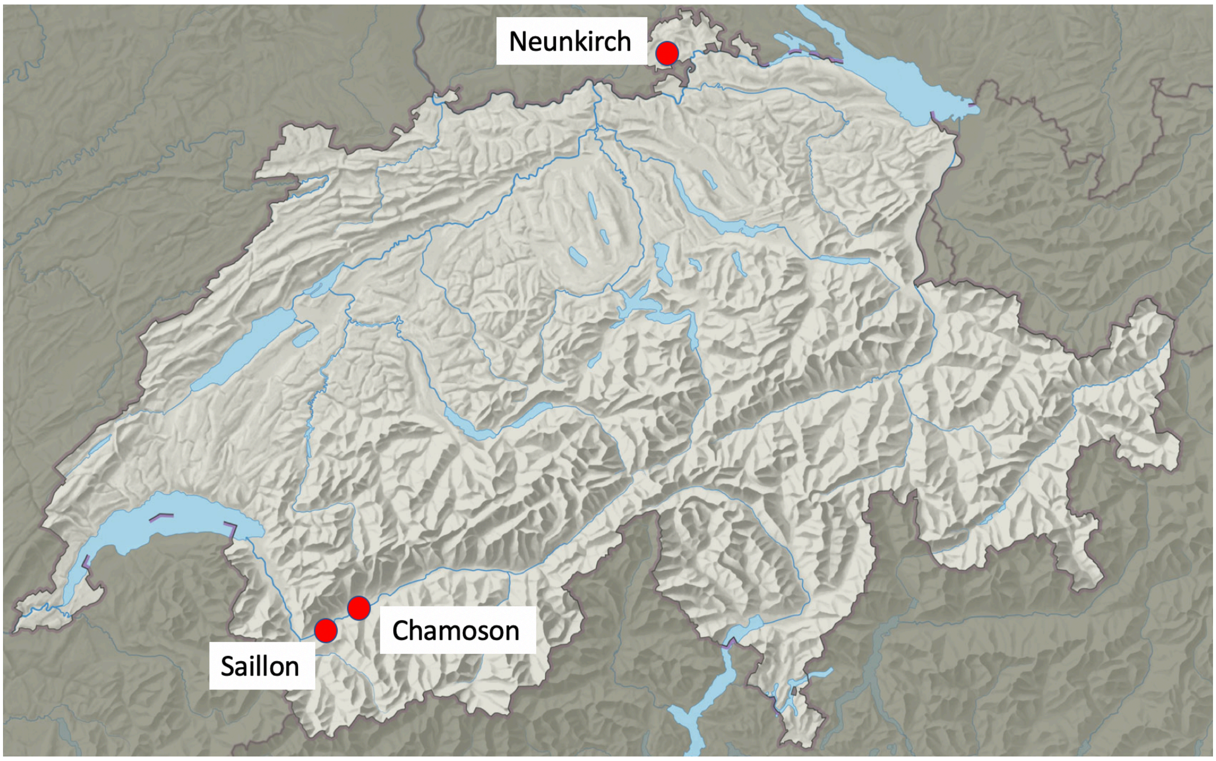

Figure S1. Map of Switzerland with the locations of three forest sites Chamoson, Neunkirch, and Saillon.

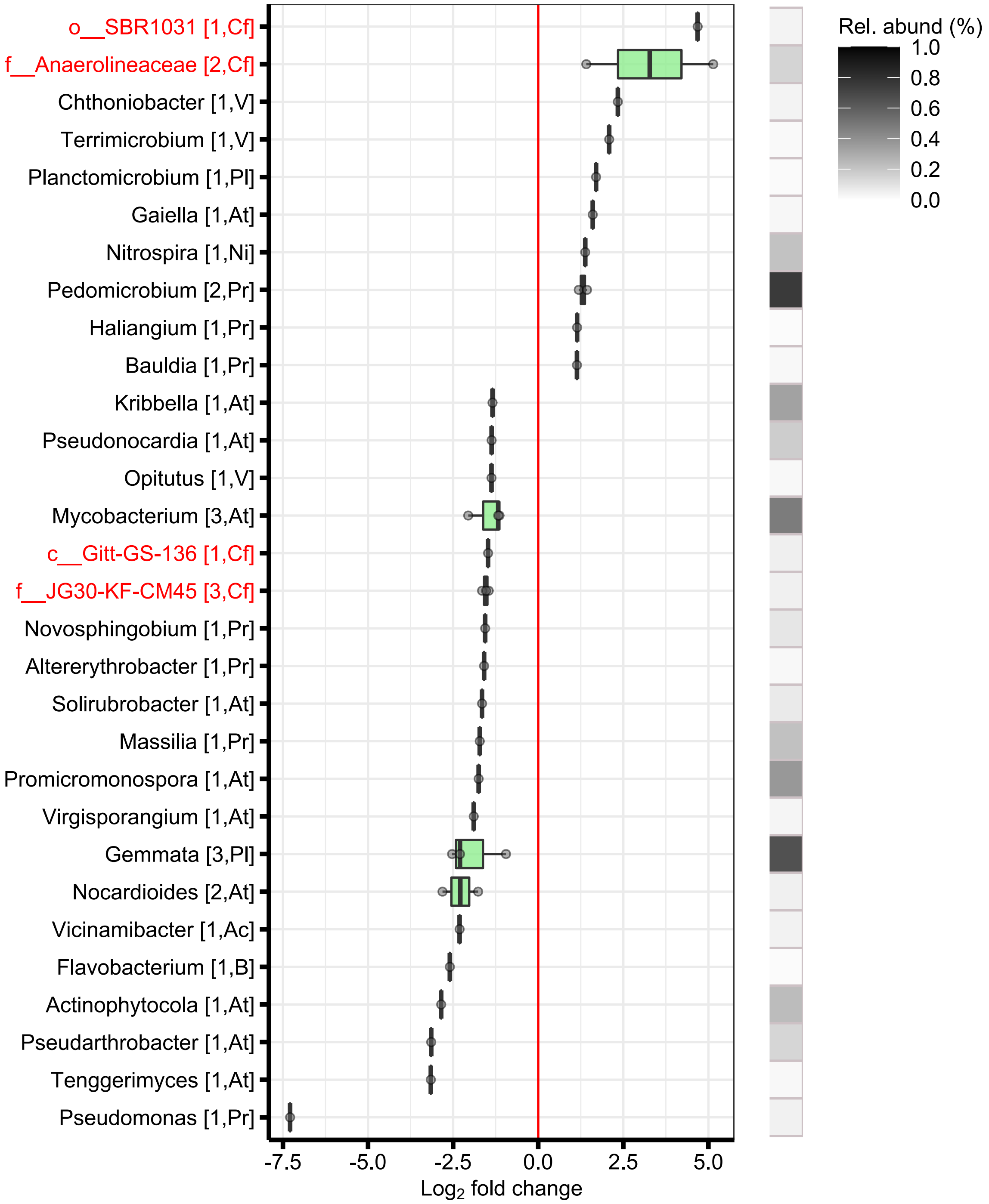

Figure S2. Differentially abundant bacteria genera of beech sites *vs*. oak sites. Shown are only significant $\log_2$-fold changes (LFC) of genera based on a significance level of $p<0.01$ after false discovery rate correction. Error bars represent standard deviation. Positive LFC values indicate a higher occurrence of the taxa in the beech sites, whereas negative LFC indicate a higher occurrence in the oak sites. Only thirty genera with the highest (positive) and lowest (negative) mean LFC values were represented. The number of differentially abundant OTUs comprised in each genus, and the phyla to which they belong are indicated in brackets. Poorly known taxa are highlighted in red. Relative abundance (%): sum of the abundance of the differentially abundant OTUs belonging to the same genera as a percentage of the total number of sequences across the compared soils. Ac = Acidobacteria; At = Actinobacteria; B = Bacteroidetes; Cf = Chloroflexi; Ni = Nitrospirae; Pl = Planctomycetes; Pr = Proteobacteria; V = Verrucomicrobia.

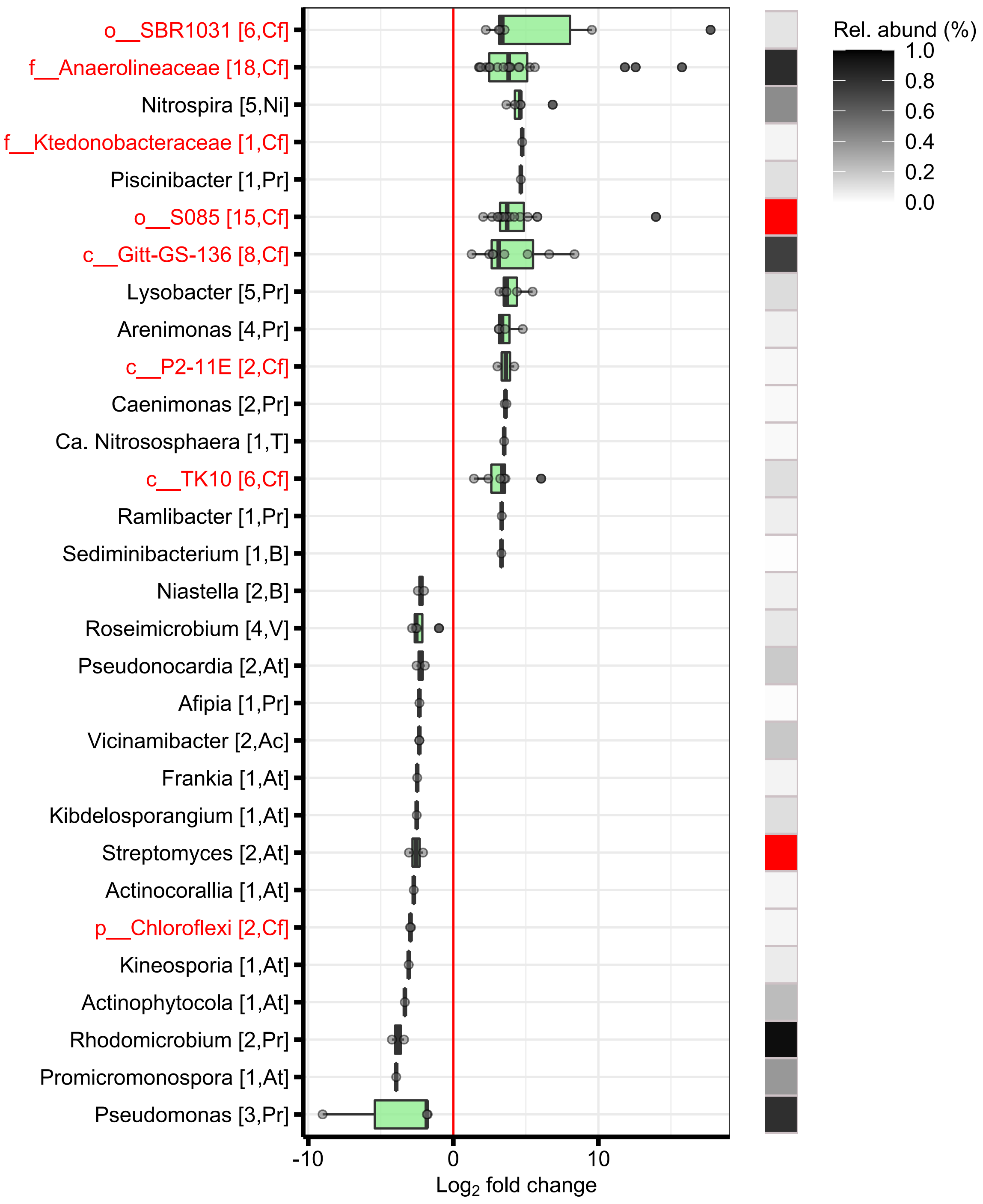

Figure S3. Differentially abundant bacteria genera of bulk soils *vs*. fine roots. Shown are only significant log_2-fold changes (LFC) of genera based on a significance level of $p<0.01$ after false discovery rate correction. Error bars represent standard deviation. Positive LFC values indicate a higher occurrence of the taxa in bulk soils, whereas negative LFC indicate a higher occurrence in fine roots. Only thirty genera with the highest (positive) and lowest (negative) mean LFC values were represented. The number of differentially abundant OTUs comprised in each genus, and the phyla to which they belong are indicated in brackets. Poorly known taxa are highlighted in red. Relative abundance (%): sum of the abundance of the differentially abundant OTUs belonging to the same genera as a percentage of the total number of sequences across the compared soils. Relative abundance depicted in red exceeds the abundance scale. Ac = Acidobacteria; At = Actinobacteria; B = Bacteroidetes; Cf = Chloroflexi; Ni = Nitrospirae; Pr = Proteobacteria; T = Thaumarchaeota; V = Verrucomicrobia.

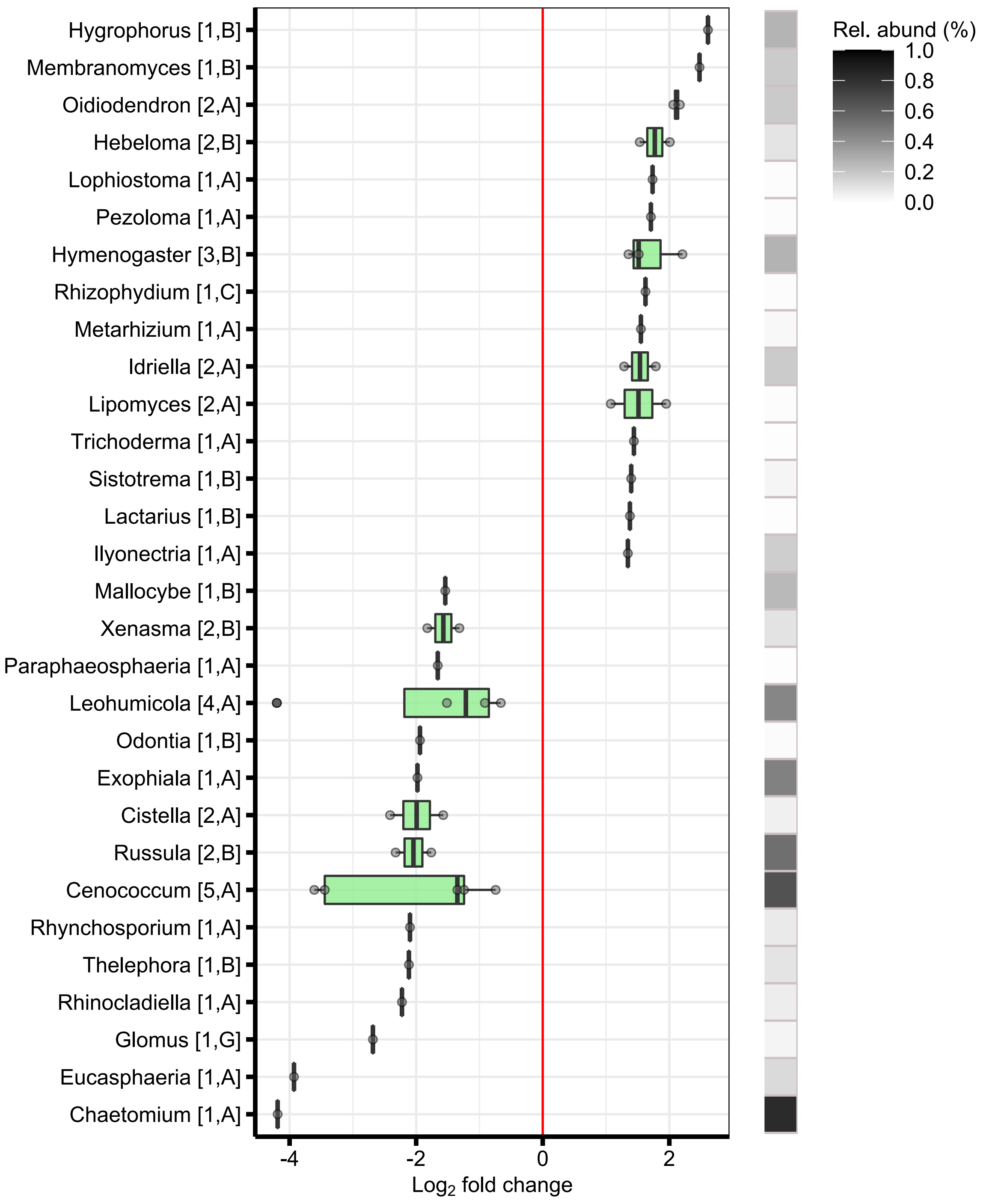

Figure S4. Differentially abundant fungal genera of beech sites *vs*. oak sites. Shown are only significant log_2-fold changes (LFC) of genera based on a significance level of $p<0.01$ after false discovery rate correction. Error bars represent standard deviation. Positive LFC values indicate a higher occurrence of the taxa in the beech sites, whereas negative LFC indicate a higher occurrence in the oak sites. Only thirty genera with the highest (positive) and lowest (negative) mean LFC values were represented. The number of differentially abundant OTUs comprised in each genus, and the phyla to which they belong are indicated in brackets. Relative abundance (%): sum of the abundance of the differentially abundant OTUs belonging to the same genera as a percentage of the total number of sequences across the compared soils. A = Ascomycota; B = Basidiomycota; C = Chytridiomycota; G = Glomeromycota.

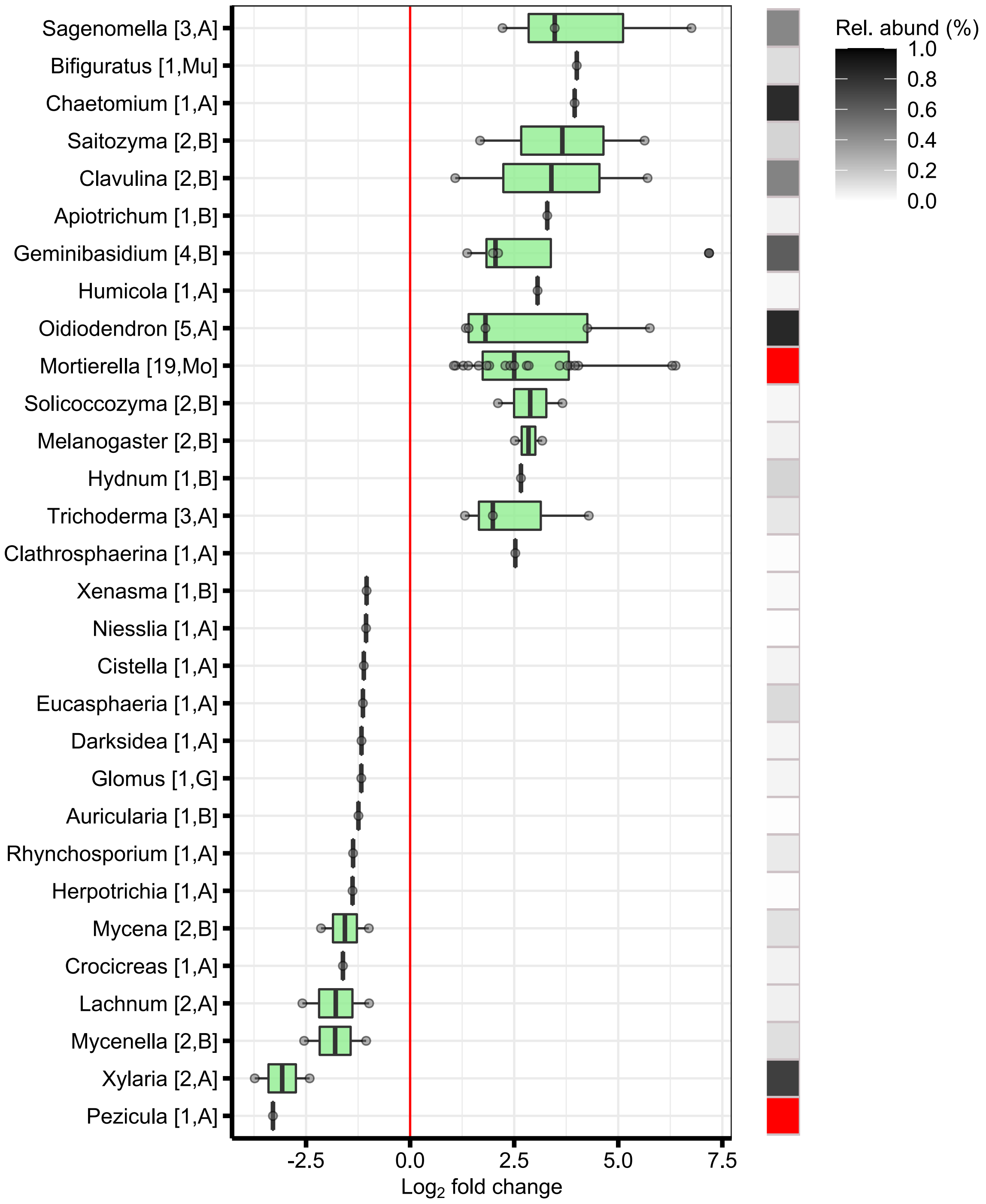

Figure S5. Differentially abundant fungal genera of bulk soils *vs*. fine roots. Shown are only significant $\log_2$-fold changes (LFC) of genera based on a significance level of $p<0.01$ after false discovery rate correction. Error bars represent standard deviation. Positive LFC values indicate a higher occurrence of the taxa in the topsoil, whereas negative LFC indicate a higher occurrence in the subsoil. Only thirty genera with the highest (positive) and lowest (negative) mean LFC values were represented. The number of differentially abundant OTUs comprised in each genus, and the phyla to which they belong are indicated in brackets. Relative abundance (%): sum of the abundance of the differentially abundant OTUs belonging to the same genera as a percentage of the total number of sequences across the compared soils. Relative abundance depicted in red exceeds the abundance scale. A = Ascomycota; B = Basidiomycota; G = Glomeromycota; Mo = Mortierellomycota; Mu = Mucoromycotina.

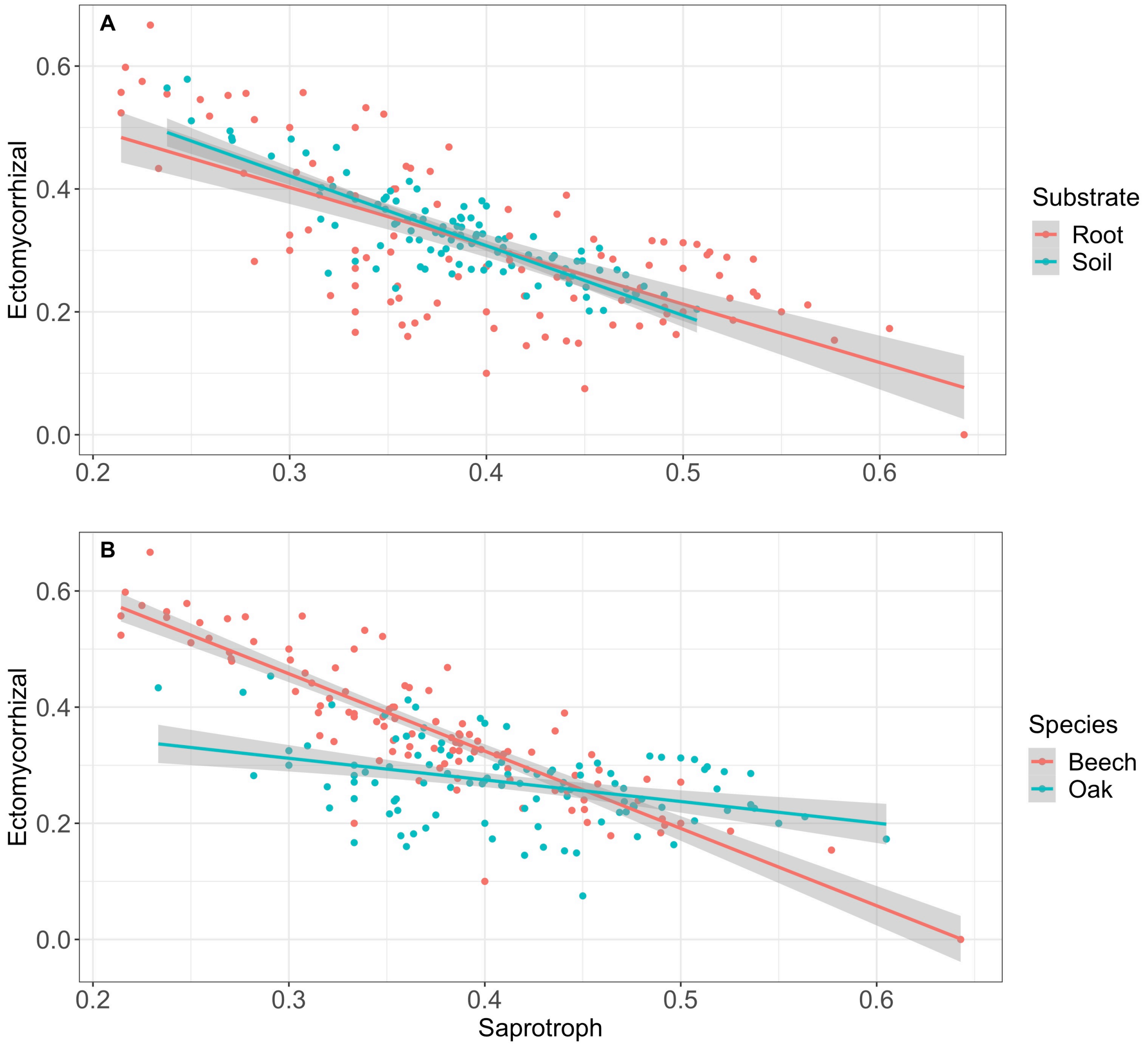

Figure S6. Comparison of the relative abundance of the two fungal guilds "*saprotrophs*" *vs.* "*ectomycorrhizal fungi*" of **(A)** bulk soils *vs.* fine roots, and **(B)** beech sites *vs.* oak sites.
